# Controlled sulfur-based engineering confers mouldability to phosphorothioate antisense oligonucleotides

**DOI:** 10.1093/nar/gkad309

**Published:** 2023-04-26

**Authors:** Vito Genna, Javier Iglesias-Fernández, Laura Reyes-Fraile, Nuria Villegas, Kevin Guckian, Punit Seth, Brad Wan, Cristina Cabrero, Montserrat Terrazas, Isabelle Brun-Heath, Carlos González, Simone Sciabola, Anabella Villalobos, Modesto Orozco

**Affiliations:** Mechanisms of Diseases, Institute for Research in Biomedicine (IRB Barcelona), The Barcelona Institute of Science and Technology, Baldiri Reixac 10-12, Barcelona 08028, Spain; NBD | Nostrum Biodiscovery, Baldiri Reixac 10, Barcelona 08028, Spain; NBD | Nostrum Biodiscovery, Baldiri Reixac 10, Barcelona 08028, Spain; Mechanisms of Diseases, Institute for Research in Biomedicine (IRB Barcelona), The Barcelona Institute of Science and Technology, Baldiri Reixac 10-12, Barcelona 08028, Spain; Mechanisms of Diseases, Institute for Research in Biomedicine (IRB Barcelona), The Barcelona Institute of Science and Technology, Baldiri Reixac 10-12, Barcelona 08028, Spain; Biogen, 225 Binney Street, Cambridge, MA 02142, USA; Ionis Pharmaceuticals Inc., 2855 Gazelle Court, Carlsbad, CA 92010, USA; Ionis Pharmaceuticals Inc., 2855 Gazelle Court, Carlsbad, CA 92010, USA; Instituto de Química Física Rocasolano, C/ Serrano 119, Madrid 28006, Spain; Mechanisms of Diseases, Institute for Research in Biomedicine (IRB Barcelona), The Barcelona Institute of Science and Technology, Baldiri Reixac 10-12, Barcelona 08028, Spain; Department of Inorganic and Organic Chemistry, Section of Organic Chemistry, IBUB, University of Barcelona, Martí i Franquès 1-11, 08028 Barcelona, Spain; Mechanisms of Diseases, Institute for Research in Biomedicine (IRB Barcelona), The Barcelona Institute of Science and Technology, Baldiri Reixac 10-12, Barcelona 08028, Spain; Instituto de Química Física Rocasolano, C/ Serrano 119, Madrid 28006, Spain; Biogen, 225 Binney Street, Cambridge, MA 02142, USA; Biogen, 225 Binney Street, Cambridge, MA 02142, USA; Mechanisms of Diseases, Institute for Research in Biomedicine (IRB Barcelona), The Barcelona Institute of Science and Technology, Baldiri Reixac 10-12, Barcelona 08028, Spain; Department of Biochemistry and Biomedicine, University of Barcelona, Barcelona 08028, Spain

## Abstract

Phosphorothioates (PS) have proven their effectiveness in the area of therapeutic oligonucleotides with applications spanning from cancer treatment to neurodegenerative disorders. Initially, PS substitution was introduced for the antisense oligonucleotides (PS ASOs) because it confers an increased nuclease resistance meanwhile ameliorates cellular uptake and *in-vivo* bioavailability. Thus, PS oligonucleotides have been elevated to a fundamental asset in the realm of gene silencing therapeutic methodologies. But, despite their wide use, little is known on the possibly different structural changes PS-substitutions may provoke in DNA·RNA hybrids. Additionally, scarce information and significant controversy exists on the role of phosphorothioate chirality in modulating PS properties. Here, through comprehensive computational investigations and experimental measurements, we shed light on the impact of PS chirality in DNA-based antisense oligonucleotides; how the different phosphorothioate diastereomers impact DNA topology, stability and flexibility to ultimately disclose *pro-Sp* S and *pro-Rp* S roles at the catalytic core of DNA Exonuclease and Human Ribonuclease H; two major obstacles in ASOs-based therapies. Altogether, our results provide full-atom and mechanistic insights on the structural aberrations PS-substitutions provoke and explain the origin of nuclease resistance PS-linkages confer to DNA·RNA hybrids; crucial information to improve current ASOs-based therapies.

## Graphical Abstract

**Graphical Abstract:**
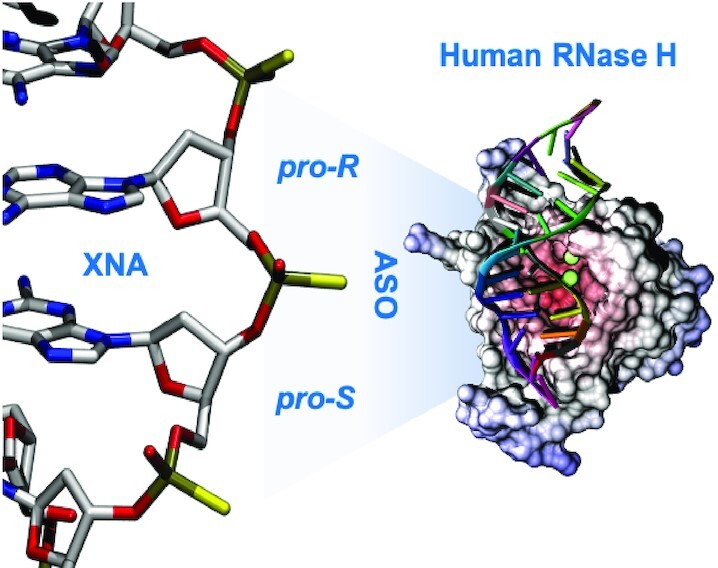
Phosphorothioate antisense oligonucleotide showing how proper stereoisomer configuration can lead to a selected XNA:RNA topology for RNase H recognition, binding and processing.

## INTRODUCTION

Antisense oligonucleotides (ASOs) are short single stranded polymers designed to recognize and interact with a target cellular RNA to disturb its normal functioning and generate a therapeutic response (1,2). Milasen (3), Fomivirsen (4). Eteplirsen (5), Golodirsen (6), Vitolarsen (7), Volanesorsen (8), Mipomersen (9), Inotersen (10) and Nusinersen (11), are only few examples of ASOs already approved by FDA and/or EMA to treat a variety of severe pathologies (12–14) and dozens of new oligonucleotide-based therapies are expected to reach the market soon. Thus, ASOs are no longer a diffuse pharmacological promise, but a real strategy to fight complex diseases. ASO pharmacological activity might be related to, at least, three different biological mechanisms: (i) steric blockage of ribosomal binding, (ii) splice-switching and (iii) RNase H-mediated degradation of target RNAs (14–17). The latest mechanism has the advantage to be catalytically-amplified as RNase H recognizes the ASO·RNA complex, degrades progressively the RNA strand, and leaves ASO intact to capture another RNA molecule and start a new functional cycle. Several studies have provided evidence that an intermediate A/B-conformation (neither DNA·DNA nor RNA·RNA) with a marked flexibility and strand asymmetry is a *conditio sine qua non* for an efficient RNase H recognition, binding and substrate degradation (18–20). As such, ASO should be based on a DNA backbone and must generate a well-structured DNA·RNA hybrid to unleash its full activity.

DNA-based ASOs can generate strong DNA·RNA hybrids, however since the single DNA strand is quickly degraded in plasma, medical use of DNA-based ASOs requires the application of protecting carriers, and/or DNA backbone modifications to reduce nuclease susceptibility and increase plasma half-life time. One of the most effective chemical strategies to avoid premature ASOs degradation is the substitution of one (or more) DNA phosphates with phosphorothioate (i.e. PS) derivative (21). This modification is suggested to do not impact DNA·RNA structure nor affect its thermostability, is well tolerated by RNase H, confers prolonged bioavailability meanwhile increases nucleic acids lipophilicity for a better cellular delivery (2,21,22). Phosphorothioates are chiral (*Rp* and *Sp* stereoisomers) and due to the racemic mixture that can be generated during the synthesis, specific chiral effects on the ASO and ASO:RNA topology cannot be evaluated. For instance, the therapeutic activity of the FDA-approved Mipomersen is, in fact, the result of the combined activities of an ensemble of more than half a million of different chemical entities (23), each of them with potentially very different bioactivities. Much room of improvement exists if clear rules on the connection between chirality and ASOs activity are established.

Obtaining a PS stereo-pure synthesis has represented a *grand challenge* for many years. First approaches were based on the use of modified polymerases, which provided stereo-pure PS-DNA derivatives for the *Rp* stereoisomer only, taking advantage of the strict requirement for chirality inversion in the conserved reaction mechanism (24–27). More recently, chemists have engineered a more versatile mechanism to obtain stereo-defined phosphorothioate DNA analogues (28–30), opening up to the possibility of synthesizing DNA-based ASOs with an exquisite (base-pair resolution) control on the phosphorothioate chirality (28–30), thus allowing for a detailed analysis of the impact of phosphorothioate stereochemistry in the properties of PS-DNA·RNA hybrids for therapeutic purposes. Using such oligos, Stec and co-workers (28,31,32) and Wan et al. (33) found a thermal stability order: phosphate > *Rp* > *Sp* for DNA·RNA hybrids, a trend that agrees with the detailed analysis performed by Iwamoto et al. (23), but that disagrees with recent studies by Ostergaard *et al.* (30) that suggested a different order of stability: *Rp* > phosphate > *Sp*. Structural studies of stereo-pure PS hybrids are scarce and limited to pure *Sp* or *Rp* hybrids. Early NMR and CD studies (24,32,34) indicated that phosphorothioate substitutions induce small topological changes in the DNA·RNA complex, but more recent NMR analysis showed that hybrid might differ in flexibility with relevant influence on their biological properties (18).

The impact of phosphorothioates on the biological activity of the DNA·RNA hybrids has been explored since the 90’s, again with controversial results. Early studies suggested that PS-DNA·RNA hybrids are susceptible to RNase H degradation, with the *Rp*-containing hybrids being as susceptible as the parent DNA·RNA hybrids (28). Opposite results were obtained by Iwamoto *et al.* (23), which found that the pure *Sp* hybrid is degraded better by RNase H than the pure *Rp* one. Through a systematic analysis of chimeric hybrids obtained by mixing *Rp* and *Sp* linkages, Iwamoto *et al.* (23) suggested that a precise combination of *Rp* and *Sp* stereochemistry can boost RNase H degradation. Wan *et al.* (33), and Ostergaard *et al.* (30) found a much complex situation where *Rp* or *Sp* can either enhance or reduce RNase H activity on the complementary RNA strand, finding very different combination of rules than those determined by Iwamoto *et al.* earlier (23). Interestingly, the impressive amount of experimental work carried out on phosphorothioate containing DNA·RNA hybrids has not been matched by a parallel effort based on theoretical studies. Considering the discrepancies between different experimental investigations, application of computational methods could provide useful atomistic and mechanical insights for a better understanding of the structural and biological impact of phosphorothioates and how this is modulated by the PS stereochemistry. Here, we present a massive computational effort supported by a variety of experimental measurements (thermodynamic analysis, CD and NMR spectroscopy), where the combination of QM/MM, Force-Field Based Molecular Dynamic Simulations (MD), enhanced sampling techniques and statistical mechanics were able to produce predictive models on the effect of phosphorothioate stereochemistry on the structure, stability, flexibility and biology of PS-DNA·RNA hybrids.

## MATERIALS AND METHODS

### Synthesis of antisense oligonucleotides (ASO)

To explore stability, structural and dynamical properties of chiral phosphorothioate (PS) linkages in the DNA strand of the DNA·RNA hybrid, we synthesized: a pure *Rp*, a pure *Sp* and two chimeras mixing *Rp* and *Sp* diastereomer and hybridized them with the complementary RNA strand forming duplexes (roman stands for unmodified DNA strand with PO linkages, underlined refers to the *Rp* linkage and italics to the *Sp* linkage: d(GTCCACGCGACG)·r(CGTCGCGTGGAC) (the pure *Rp* hybrid); d(*GTCCACGCGACG*)· r(CGTCGCGTGGAC) (the pure *Sp* hybrid); d(*GTCCA*CGCGACG)· r(CGTCGCGTGGAC) (the *SpRp* hybrid) and the d(GTCCA*CGCGACG*)· r(CGTCGCGTGGAC) (the *RpSp* hybrid). As a control we synthesized also the unmodified DNA strands generating the canonical hybrids d(GTCCACGCGACG)· r(CGTCGCGTGGAC). Oligonucleotides were synthesized on an ABI 394 DNA/RNA synthesizer (2 μmol scale) using polystyrene-based NittoPhase Unylinker support (405 μmol/g). Oxazaphospholidine (OAP) monomers were prepared in a manner similar to that described by Wada et al. (35). 5-Methylcytosine was used during the synthesis of the PS nucleic acids herein investigated. OAP monomers were prepared at 0.2 M in MeCN:toluene 1:1 (v/v) + 2.5% pyridine. Chiral PS oligonucleotides were synthesized using 3% dichloroacetic acid in dichloromethane for deblocking, 2.0 M *N*-(cyanomethyl)pyrrolidinium trifluoromethanesulfonate (CMPT) in acetonitrile (MeCN) as activator, 10% acetic anhydride in tetrahydrofuran (THF) and 10% *N*-methylimidazole in THF/pyridine for capping and 0.1 M xanthane hydride in pyridine:MeCN 3:2 (v:v) for thiolation. After conclusion of the synthesis the oligo was cleaved from the solid support and the remaining protecting groups were removed by treating with concentrated aqueous ammonia at 85°C for 2 h. Oligonucleotides were purified by ion-exchange chromatography using aqueous buffers A: 50 mM NaOH and B: 50 mM NaOH and 2.5 M NaCl. The DMT group was removed on-column by treatment with 6% dichloroacetic acid in water. Pure fractions were desalted on a C18 reverse phase column, eluted in 50% acetonitrile in water (v:v) and lyophilized. Purity and mass of oligonucleotides was determined using ion-pair LCMS.

**Table:** 

Ionis #	Sequence (5′ → 3′)	amt	MW calc	MW found	UV purity	FL
1559301	*GTCCACGCGAC*G	2.1	3878.22	3877.41	94.98	86.72
1559302	GTCCACGCGACG	3.0	3878.22	3877.41	95.41	86.48
1559303	*GTCCA* CGCGACG	1.5	3878.22	3877.44	93.58	85.12
1559304	GTCCA *CGCGAC*G	3.5	3878.22	3877.44	96.43	88.78

Underlined = *Rp* PS linkage, italic = *Sp* PS linkage, roman = native deoxynucleotide.

### UV-monitored thermal denaturation studies

Absorbance versus temperature curves of DNA·RNA hybrids were measured at 1 μM, 2 μM, 5 μM, 20 μM and 50 μM oligonucleotide concentration in 10 mM sodium phosphate buffer (pH 7.4) containing 150 mM NaCl. Experiments were performed using 1 cm (for 1, 2 and 5 μM oligonucleotide concentration) and 1 mm (for 20 μM and 50 μM oligonucleotide concentration) path length quartz cells on a Varian-Cary-100 spectrophotometer equipped with thermoprogrammer. The samples were heated to 95°C, allowed to slowly cool to 12°C, and then warmed during the denaturation experiments at a rate of 0.5°C*/*min to 100°C, monitoring absorbance at 260 nm. Melting temperatures and associated folding thermodynamics were obtained by three different approaches as described by Bevilacqua and coworkers and implemented in the MeltR program (https://github.com/JPSieg/MeltR; see Supplementary Methods) getting in all the cases very similar values, which actually are also very close to those obtained by a standard use of van’t Hoff equation (data not shown).

### CD measurements

CD spectra (200–320 nm with a 100 nm min^−1^ scan rate) were recorded at room temperature on a Jasco J-810 spectropolarimeter under the same buffer conditions as for UV melting curves and at 2 μM oligonucleotide concentration.

### NMR experiments

Samples of the four thiophosphates hybrids (distinguishing the chirality of the residues: all *Rp*, *RpSp*, *SpRp* and all *Sp*) were suspended in 300μl of either D_2_O or 9:1 H_2_O/D_2_O in sodium phosphate buffer (100 mM NaCl and 25 mM sodium phosphate, pH 7). All NMR spectra were acquired in Bruker Avance spectrometers operating at 600 and 800 MHz and processed with TOPSPIN software. ^1^H melting experiments, DQF-COSY, TOCSY and NOESY experiments were recorded in both D_2_O and H_2_O/D_2_O 9:1. The NOESY spectra were acquired with mixing times of 150 and 250 ms, and the TOCSY spectra were recorded with standard MLEV-17 spin-lock sequence, and 80 ms mixing time. For 2D experiments in H_2_O, water suppression was achieved by including a WATERGATE module in the pulse sequence prior to acquisition. Two-dimensional experiments in D_2_O were carried out at temperatures ranging from 25°C with pre-saturation to suppress the residual H_2_O signal, whereas spectra in H_2_O were recorded at 5°C to reduce the exchange with water. The spectral analysis program Sparky (D.K. TD Goddard and D.G. Kneller, SPARKY v3, University of California, San Francisco) was used for semiautomatic assignment of the NOESY cross-peaks and quantitative evaluation of the nuclear overhauser effect (NOE) intensities.

### Model building of nucleic acids

Starting conformations of the four naked DNA:RNA hybrids duplexes with different thiophosphate stereoisomers on DNA strand were built from Drew-Dickerson dodecamer (PDBid 1BNA) structure that provided the appropriated backbone geometry for the subsequent sequence-modeling (36), as already done in our previous studies (37). Hence, the starting canonical B-conformation has been then modeled to obtain the investigated 5′-GTCCACGCGACG-3′ sequence. Sequence modeling has been performed by means of Amber package (38). The four considered system show a 100% *Sp* [PO_3_S]^3−^ isomer namely *Sp*, 100% *Rp* [PO_3_S]^3−^ isomer namely *Rp*, 50/50% *Sp*/*Rp* with *Sp* constituting the first six nucleotides in 5′-3′ direction and namely *SpRp* and 50/50% *Rp/Sp* with *Rp* constituting the first six nucleotides in 5′-3′ direction and namely *RpSp*. For comparison, the same nucleotide sequences with native DNA·DNA, DNA·RNA and RNA·RNA were build and simulated. Parmbsc1 force-field was used to describe DNA interactions (39–41), while RNA was described using recent RNA force-field published by Tan, D. *et al.*; one of the latest developments in RNA-force fields (42–44).

### Parametrization of phosphorothioates

Free-Energy surface associated to variation of the α/ζ angles was built by means of hybrid QM/MM well-tempered metadynamics till convergence (Supplementary Figure S1). The dinucleotide was represented at the DFTB3 level of theory, while ions and solvent were represented as in production MD simulations (see below). QM/MM calculations were performed with the Amber and Terachem software (38,45). Reference DFT surfaces (Supplementary Figure S1) were used to refine torsional parameters as described elsewhere (46). Amber Parameters (frcmod and library) of phosphorothioate can be downloaded from https://mmb.irbbarcelona.org/NAFlex/Vito/phosphorothioate.frcmod and https://mmb.irbbarcelona.org/NAFlex/Vito/phosphorothioate.lib, respectively.

### Protein-complexed nucleic acids

To dissect the effect of the different *Rp* and *Sp* thiophosphate stereoisomers when complexed to nucleic acid processing enzymes, we studied two well-known and widely characterized systems; the highly-processive 5′→3′ Klenow Fragment exodeoxyribonuclease and the non-sequence specific Human RNAse H. The Klenow Fragment exodeoxyribonuclease complex was generated starting from the X-ray structure (PDBid 1KFS, 2.1 Å resolution) (47). RNase H calculations are based on the crystallographic structure of the Human RNAse-H in complex with the hybrid RNA·DNA substrate as in the D210N mutated conformation (PDBid 2QKK, 3.2 Å resolution) (48). Since the D210N-mutant enzyme is inactive, N210 has been reverted to D210 in order to reproduce the wild-type and catalytically active conformation, as done in several other studies (49–53). Focus has been put on the structural arrangement of the bi-nuclear active site which, by containing the well-known DEDD-motif, must respect a well-shaped active site (two-metal-ion centered) in order to permit correct substrate posing and subsequent catalysis (25–27,54–57). Furthermore, additional spots for bad contacts/clashes were visually analyzed along the interaction area between protein and RNA·DNA substrate (i.e. positively charged cleft). Proteins were represented using the all-atom AMBER/FF14SB (58) force-field.

### Alchemical free energy calculations

MD-based alchemical free energy calculations require the generation of hybrid structure/topology. A successful single topology approach to construct hybrid residues was reported by Seeliger *et al.* (59) and has been here applied as already done in previous works (60–62). The reversible work associated to the *Sp* <–> *Rp* interconversions were determined for single stranded DNAs and DNA·RNA hybrids. Works were combined using standard thermodynamic cycles to obtain variation of the associated free energy related to the changes from phosphate to thiophosphates and from *Sp* stereoisomer to the other (Figure 3). Calculation was repeated for all the 16 dinucleotide steps (also embedded in a common dodecamer with Drew Dickerson sequence) (36) to check for sequence effects. Furthermore, to explore for potential cooperative (or anti-cooperative) effects, 1–3–5–7–9–11 mutations were done sequentially using again the same DNA·RNA hybrid dodecamer. Reversible works associated to phosphate changes in the single stranded and duplex structures were determined from thermodynamic integration using 11 double-wide sampling windows, each simulated for 10 ns. The same alchemical protocol was used to check the change in association free energy of a hybrid when the chirality of PS which are in close contact with the active domain of RNAse H. We explore the changes in free energy associated to the 2^3^ potential combinations or *Rp* and *Sp* chirality in the phosphates in close contact with the protein in the DNA·RNA complex as well as the same groups in the unbound state, subtracting both numbers to derive the changes in association free energy (Supplementary Figure S2).

### Molecular dynamics simulations setting and protocol

All the systems were solvated with waters and neutralized with Na^+^ adding 100 mM additional NaCl. Water molecules were represented by the TIP3P model (63), while ions were modeled by using Joung-Cheatham parameters for monovalent ions and Li/Merz parameters for Mg^2+^, respectively (64,65). The size of the final triclinic box was approximately ∼60 Å × 100 Å × 60 Å for freely dispersed nucleic acids and ∼85 Å × 90 Å × 100 Å for RNase H and Klenow Fragment exonuclease complexes. Simulation systems were optimized and slowly heated-up and equilibrated for 50 ns prior to production that has been extended for 1 μs in the isothermal isobaric ensemble (NPT; *T* = 310 K and *P* = 1 atm). Long-range electrostatic interactions were calculated with the particle mesh Ewald method (PME) with a real space cut-off of 12 Å and periodic boundary conditions in the three directions of Cartesian space were used (66). Constant temperature was imposed using Langevin dynamics (67) with a damping coefficient of 1 ps, while pressure was maintained with Langevin-Piston dynamics (68) with a 200 fs decay period and a 50 fs time constant. LINCS (69) was used to maintain covalent bonds at equilibrium distance, allowing the use of 2 fs integration step. All MD simulations were performed using *GRO*ningen *MA*chine for *C*hemical *S*imulations (GROMACS) 2020 code, collecting data every 5 ps (70). To improve statistics, 5 replicas for each of the analyzed system have been generated resulting in an overall simulated time of ∼21 μs (1 μs per system). Analysis were carried out using GROMACS analysis tools, VMD 1.9 Software (71), Curves+ (72) and NAFlex and BIGNAsim analysis tools (73,74). Essential dynamics studies were done from principal components obtained from the diagonalization of the associated covariance matrix as described elsewhere (75). Trajectories were stored in our BIGNAsim database (74) following FAIR data standards as described elsewhere (76).

## RESULTS

### Impact of PS chirality on the topology and mechanics of DNA·RNA hybrid

**Figure 1.**
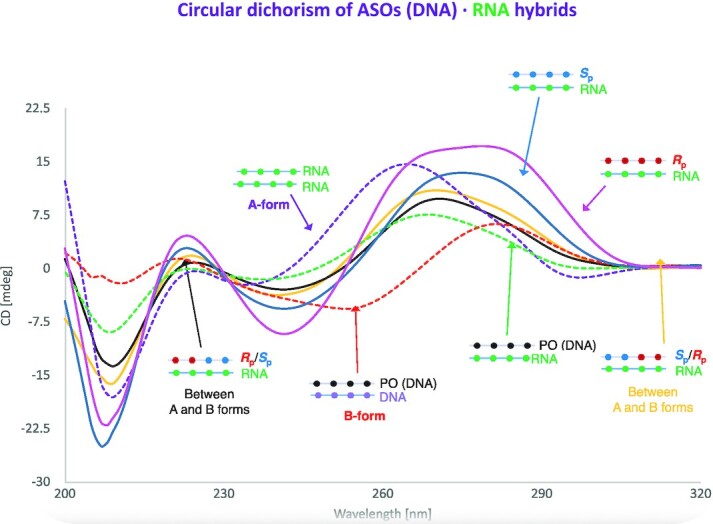
Circular dichroism spectra of the different DNA·RNA duplexes here investigated. Reference conformations are reported by dashed lines: the red dashed one indicates the canonical B-conformation of DNA·DNA duplex, the purple dashed one describes the canonical A-conformation typical of the RNA·RNA duplex while the dashed green line represents the non-A/non-B conformation associated with the hybrid DNA·RNA duplexes. *Rp*DNA·RNA is shown as pink line, *Sp*DNA·RNA in blue, *SpRp*DNA·RNA in yellow and *RpSp*DNA·RNA in black. See Methods for the experimental conditions.

Hybrids containing phosphorothioates (PS) in the DNA are characterized by CD spectra that clearly differ from that typical of the RNA·RNA (i.e. A-conformation featured by a maximum peak at 270 nm and a shallow lower peak) as well as from that identifying the DNA·DNA (i.e. B-conformation described by a maximum peak at ∼275 nm and a lower peak located at ∼250 nm) (Figure 1). Also, PS-containing hybrids show CD spectra that also differ from those of the RNA·DNA duplexes that fall somehow in the middle (i.e. non-A nor B-conformation, Figure 1). Overall hybrids are closer to the A- than the B-conformation, with non-negligible differences depending on the chirality of the PS linkages present in the DNA backbone. Indeed, the *Sp* hybrid (PS presenting pro-*Sp* S substitution; Figure 1) closely matches A-like conformation, while the *Rp* one (PS with the pro-*Rp* S configuration) shows, instead, a more A/B intermediate topology (Figure 1).

**Figure 2.**
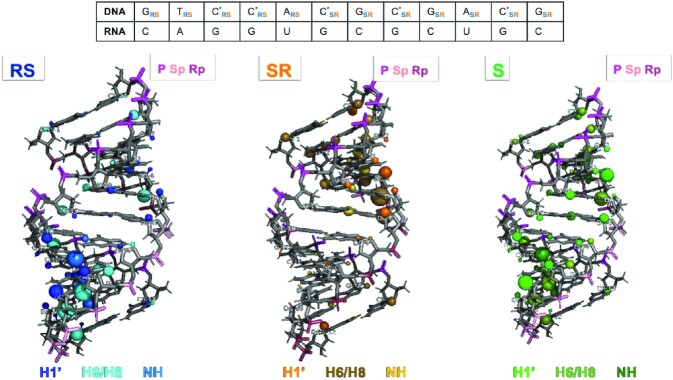
Representation of chemical shift differences between the *RpSp* (left), *SpRp* (center) and *Sp* (right) with respect to the *Rp* hybrid duplex. Nucleobase protons are shown in blue and sugar protons in cyan, light orange and light green. The size of the spheres represents the chemical shift differences (small, medium or large mean differences of 0.04–0.1, 0.1–0.2 or >0.2 ppm, respectively). RNA phosphates are shown in dark red; PS DNA linkages are shown in magenta (*Rp*) or pink (*Sp*).

The variation of imino protons signals at increasing temperature from—5°C to 45°C—confirmed that the four studied duplex hybrids are stable under the NMR experimental conditions (Supplementary Figure S3). Sequential assignments of proton resonances were conducted following standard methods for right-handed, double-stranded nucleic acids. The assignment pathways in the base-H1’ and base-methyl regions is illustrated for the *Rp*DNA·RNA duplex in Supplementary Figure S4. Exchangeable protons were assigned with the NOESY spectra recorded in H_2_O (Supplementary Figure S5A). Most of the labile protons were assigned following standard methods, except some amino resonances of the terminal residues that were not detected, thus suggesting some fraying in terminal base pairs. The cross-peak patterns observed for the exchangeable protons indicate that all bases are forming Watson-Crick pairs (Supplementary Figure S5); thus, further confirming the presence of a well-structured double helix formed by each of the analysed system. Although the proton spectra of the four duplexes are overall similar, very clear differences can be observed, confirming that chirality at the PS linkages induce significant structural changes (Supplementary Figures S5 and S6). This is well shown by the chemical shifts’ differences for the same proton in the four duplexes. (Supplementary Figures S5–S7). In chimeric *RpSp* and *SpRp* hybrids we found a nice conservation in the signals with respect to the stereo-pure *Sp* and *Rp* hybrids in the region of the same chirality (Figure 2), with the largest differences being located in the boundary region between the two *Rp/Sp* tracts. This suggests locality in the effect of phosphorothioate chirality.

### Molecular dynamic simulations

We performed extensive MD simulations of the duplexes under investigation. Duplexes were stable along the simulated time-scale and the canonical Watson-Crick hydrogen-bond pattern was maintained throughout the whole simulated timescale. The phosphorothioate-containing hybrids show an intermediate A/B conformation closer, in general, to the A-form than to the B-one (see Table 1). Indeed, in agreement with CD spectra (Figure 1), the hybrid with *Rp* linkages shows the most intermediate A-/B-conformation while the presence of *Sp* linkages shifts the overall structure geometry towards the canonical A-form.

**Table 1.** RMSd, in Å, calculated for the system under investigation. White background indicates RMSd values calculated starting from the equilibrated snapshots of our MD simulations. Green background indicates RMSd values calculated on the average structure representing MD trajectories. Green boxes report the value obtained comparing single snapshots represented by average structures

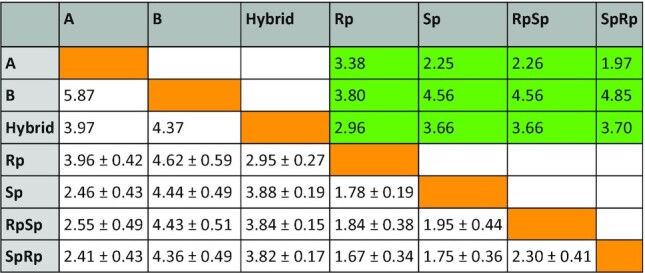

Further analysis of the MD trajectories of the chimeric hybrids containing phosphorothioate in the DNA backbone, with *Sp*/*Rp* ratio of ∼50%, indicate that a local rearrangement occurs right at the interface between *Sp* and *Rp* (positions 6 and 7) linkages. In this region, we observed ζ-dihedral angle shifting (from ∼−100° to ∼−50°, Supplementary Figure S8) that populates almost the ∼65% of the propagated trajectory. This effect is visible for the thiophosphate group lying just at the interface of the chimeric *SpRp* hybrids. The same torsional returns to its normal value in the surrounding nucleotides. All these findings agree with the existence of small structural distortion at the junction between *Sp* and *Rp* stereoisomers in chimeric hybrids, as also highlighted by NMR experiments.

### Essential dynamics of phosphorothioate hybrids

As already described elsewhere (77–82), principal component analysis (PCA) was used to describe the essential deformation pattern of the different systems under investigation. For the sake of clarity and based on previous works (77–82), we focused our analysis on the first 10 modes, which describe more than 80% of conformational space sampled by the hybrids (Supplementary Figure S9). Analysis and comparison of eigenvalues indicate (78,83) RNA·RNA as the most rigid duplex, and DNA·DNA as the most flexible one. The natural DNA·RNA hybrid shows a different pattern of flexibility, similar to that of DNA·DNA for the lower modes and approaching the RNA·RNA for the higher ones. PS hybrids show also an intermediate global flexibility, but are in general more rigid than the natural DNA·RNA ones, with no impact of the PS chirality (Supplementary Figure S9). Analysis of the deformation modes using Hess metrics (75), indicates (see Supplementary Table S1) that: (i) trajectories are well converged (self-similarity indexes > 0.9, Supplementary Table S1); (ii) DNA·DNA and RNA·RNA essential deformations are quite different (similarity index around 0.5); (iii) DNA·RNA deformation pattern is intermediate between those of pure homopolymers; (iv) the presence of PS linkages deforms the nature of the hybrid essential deformation pattern; (v) the pure *Rp* hybrid displays a pattern of flexibility closer to the native DNA·RNA, while the pure *Sp* hybrid shows a deformation pattern closer to that of a pure RNA duplex (see Supplementary Table S1).

### Phosphorothioate chirality and thermal stability

**Figure 3.**
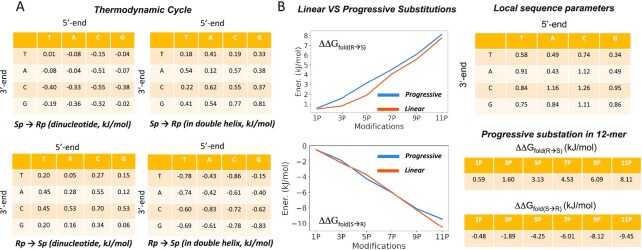
(**A**) Thermodynamic cycle calculated to investigate energetically *Sp* to *Rp* (ad *vice-versa*) interconversion. (**B**) panels show progressive substitution while tables on the right report detailed free energy values (in kJ/mol). The standard errors associated to values in panel A are always ≤0.004 kJ/mol. Those at the right have associated standard errors which are typically around 1–5% of the estimated value.

To energetically dissect pro-*Sp* and pro-*Rp* phosphorothioate (PS) substitutions in the DNA strand of the PS DNA·RNA hybrid, we performed accurate alchemical *Sp*/*Rp* transformations in all the 16 different dinucleotide combinations as well as in a common hybrid duplex containing the 16 different bases. Free energy calculations were performed in both directions (*Rp*→*Sp* and *Sp*→*Rp*) to obtain an estimate of the associated statistical errors. Figure 3A indicates an excellent convergence of the individual values for both single strands and duplexes. Combining these estimates with standard thermodynamic cycles, we obtained a sequence-dependent predictor of the impact of *Sp*/*Rp* chirality on the stability of the hybrid duplexes. Results in Figure 3 indicate that the *Rp* stereoisomer confers, overall, more stability to the duplex than the *Sp* stereoisomer. Interestingly, our calculations also highlight a small (but non-negligible) sequence-dependence stability, as the energy gain passing from *Sp* to *Rp* chirality in one linkage can variate from 0.3 up to 1.3 kJ/mol. An unsolved question on the relative stability of *Sp/Rp* linkages is whether or not the stability changes are additive or rather show a certain positive or negative cooperativity. To solve this question, we performed progressive mutations (from 1 to 11 phosphate groups of the dodecamer) on a hybrid duplex comparing the stability predictions with those obtained by simple addition of individual linkages (Figure 3).

Finally, we explored the change in stability associated to the PS → PO linkage transformation, thus reverting into native nucleic acids form. Towards this end, we repeated the same protocol and methodologies reported above but mutating from *Rp* → O which, combined with the *Sp*/*Rp* information, allow us to derive sequence-specific descriptors of the stability change produced by substituting one phosphate linkage with a phosphorothioate one (of either *Rp* or *Sp* chirality).

**Figure 4.**
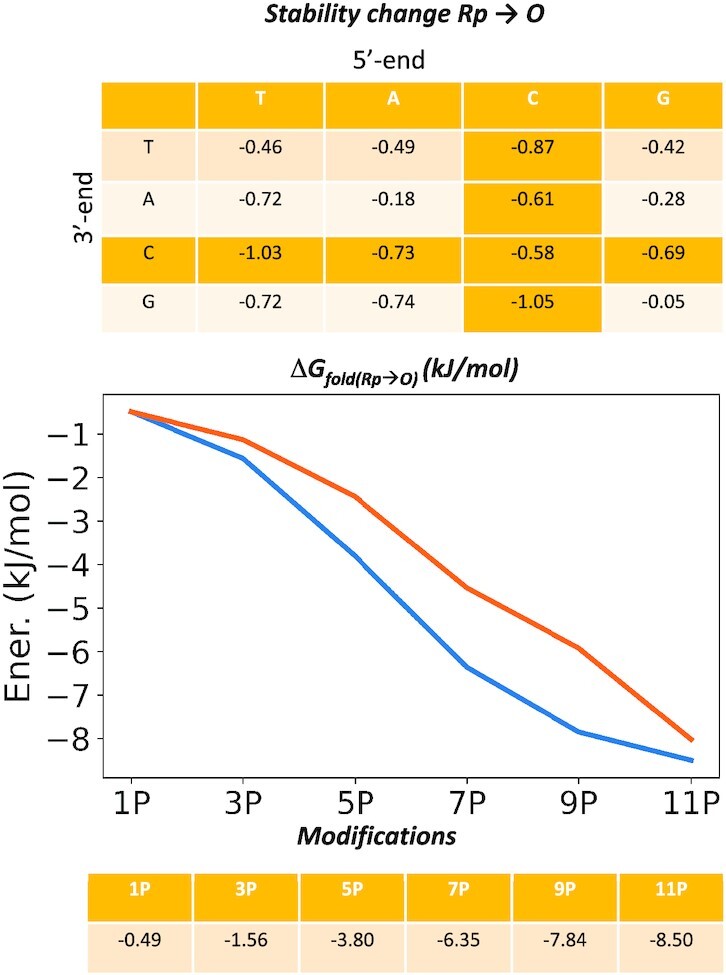
Thermodynamic cycle calculated to investigate energetically Rp → O alchemical transformation and relative free energy values. Averages shown in panel A have an associated standard error ≤0.004 kJ/mol. Those at the right have associated standard errors which are typically around 2–6% of the estimated value.

Values in Figure 4 clearly indicate that for all the investigated cases the natural phosphate linker confers more stability to the hybrid than the most stable phosphorothioate linker (i.e. *Rp*); with a ΔG spanning from −0.49 to −8.5 kJ/mol. By combining results of our experiments (Figures 3 and 4) we can derive an order of stability that is: PO > *Rp* > *Sp*. To validate this ordering, we determined experimental melting temperatures of a series of hybrids containing different combinations of *Rp*/*Sp* linkages (Table 2). Melting temperature (Tm) data follow a general decreasing trend with PO > *Rp* > *Sp* with *RpSp* ≈ *SpRp* falling in between *Rp* and *Sp*, as expected from additivity effect previously observed. The same order is obtained by inspecting relative folding free energies derived from thermodynamics analysis of the melting curves (see Materials and Methods). In fact, agreement between theoretical and experimental differential free energies is excellent, supporting the reliability of our calculations and the accuracy of our theoretical predictor.

**Table 2.** Comparison of relative Tm and folding free energies for different hybrids determined experimentally and relative free energy estimated from our mesoscopic model. Values are compared to the *pro-Rp* S-substituted system. Roman means standard phosphate linkages, underlined characters stand for the R PS linker (*Rp*) and italics refers to S PS linker (*Sp*). ΔTm are in degrees and free energy values are in kJ/mol before the slash and kcal/mol after the slash. All values are referred to the corresponding all *Rp* hybrid of the same sequence (31). Error of our predictor falls in the range of 10%. Absolute melting temperatures for the hybrids considered here and the associated parameters for folding thermodynamics are shown in Supplementary Table S2

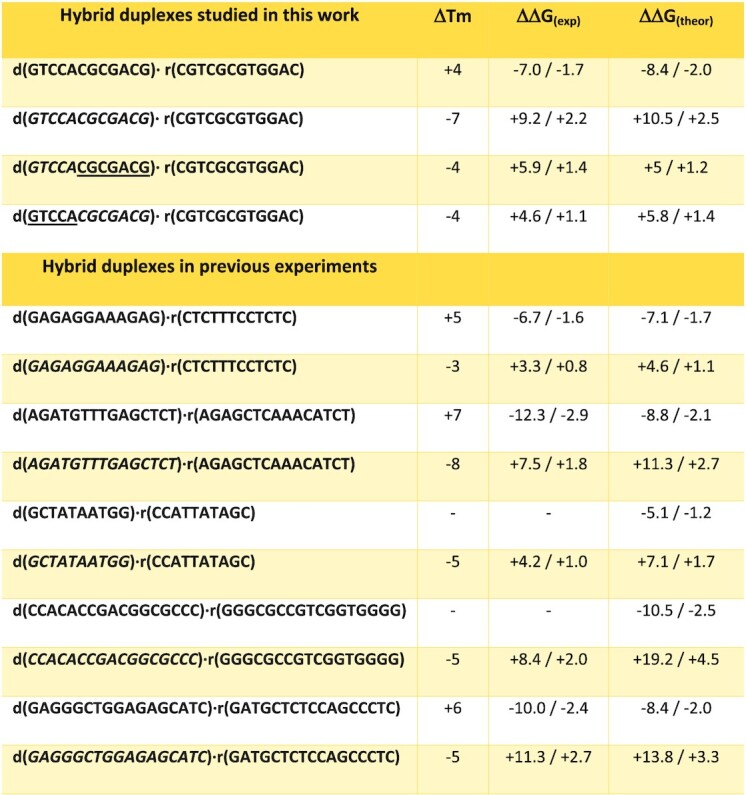

### Impact of phosphorothioates on enzymatic activity

**Figure 5.**
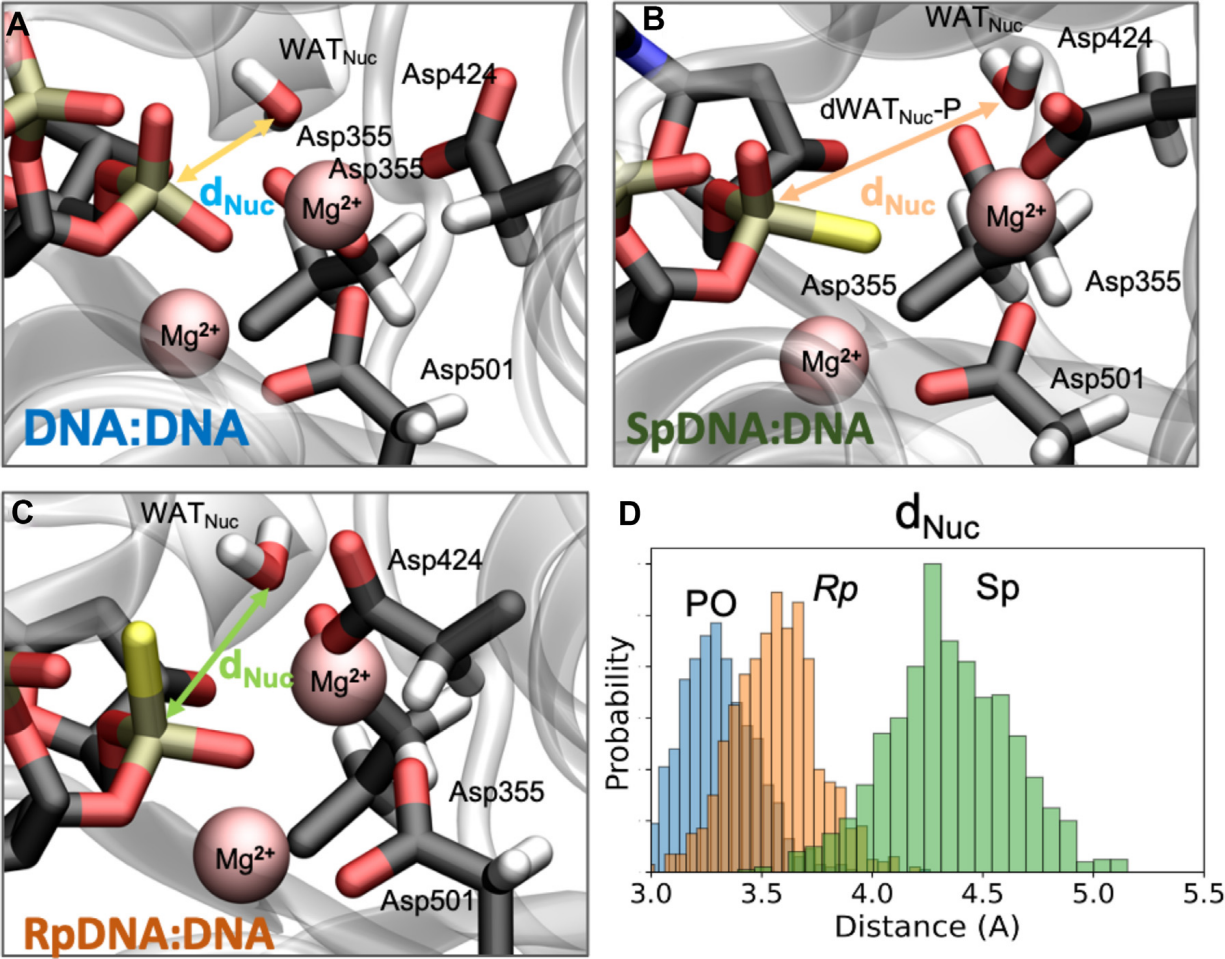
Active site of Klenow fragment exonuclease complexed with DNA·DNA (**A**) *Sp*DNA·DNA (**B**) and *Rp*DNA·DNA (**C**). The two-Mg^2+^ centered active site is shown. The nucleophilic water molecule (WAT_Nuc_) is coordinated to one of the Mg^2+^ ion (in pink). The reactive distance (i.e. *d*_Nuc_) between nucleophile (i.e. WAT_Nuc_) and electrophile (i.e. P atom) is reported and taken as a key geometrical description to evaluate the correct geometry of Exonuclease reactive active site, a well-known arrangement (25). (**D**) Frequency Distribution of *d*_Nuc_ length calculated for the different systems under investigation. Three additional replicas have been performed to explore the reproducibility of these findings. The corresponding histograms nearly overlap and are not shown for clarity reasons, but are available upon request.

We performed extensive calculation of two well-known enzymes: (i) the 3′→5′ exonuclease activity of the Klenow Fragment (PDBid 1KFS) (47,84,85) as a model of nucleases that would degrade DNA-based ASOs and (ii) the human RNase H1 (2QKK) (48), a major player in antisense activity being responsible of digesting the RNA strand of the DNA·RNA hybrid. Both systems were simulated firstly *via* force-field based MD simulations (∼1 μs each, see Materials and Methods section) and one well-equilibrated snapshot was used for further structural refinements performed by means of hybrid Quantum-Mechanics/Molecular Mechanics (QM/MM) calculations. The Exonuclease catalytic and bi-nuclear active site is defined by the so-called DEDD-motif (25–27) that coordinates two Mg^2+^ ions (indeed the crystallographic Zn^2+^ has been replaced by the catalytically active Mg^2+^). In the simulated wild-type, thus containing a natural ssDNA, the catalytic core is structurally preserved along all the trajectory, with the active water (responsible of the nucleophilic attack after its deprotonation) (25,26,86) perfectly positioned to attack the phosphate group (Figure 5B). Key structural descriptors such as the octahedral magnesium coordination geometry, the internuclear Mg^2+^–Mg^2+^ distance of ∼4.2 ± 0.39 Å and a reaction coordinate length (O_wat_-P, d) of 3.22 ± 0.64 Å report for a well-arranged system in a ready-to-react setting, as already observed earlier (25,26,55,87).

By taking advantage from this stable and equilibrated control system, we have built up a variant where the DNA strand under cleavage contains the phosphorothioate groups. Interestingly, we noted that when S atom replaces the *pro-Rp* oxygen of the scissile phosphate (so constituting the *Rp* DNA·RNA system) the catalytic water molecules falls 0.5 Å apart due to the longer P-S bond and the larger S radius. Concomitantly, the Mg^2+^–Mg^2+^ distance, a key indicator of a well-arranged pre-reactive complex, extends up to a value of ∼4.8 Å thus reflecting a less prone-to-react enzymatic configuration. On the other side, for the system where the DNA strand was engineered with *Sp* linkages, that is S replacing the *pro-Sp* oxygen of the scissile phosphate group, we observed (after only ∼270 ns) a partial unfolding of the active site provoked by the increasing Mg^2+^–Mg^2+^ distance that gets to a value up to 5.3 Å (in one of the replicas more than 6 Å), incompatible with catalysis (see Supplementary Figure S10). To validate theoretical results, we explored the ‘in vivo’ stability of single stranded DNA and the different phosphorothioate derivatives in fetal bovine serum, human serum, DNase I, Snake Venom Phosphodiesterase 1, and the Klenow Fragment Exonuclease (herein reported). Results shown in Supplementary Figures S11 and S12 fully confirm the theoretical findings reported here.

**Figure 6.**
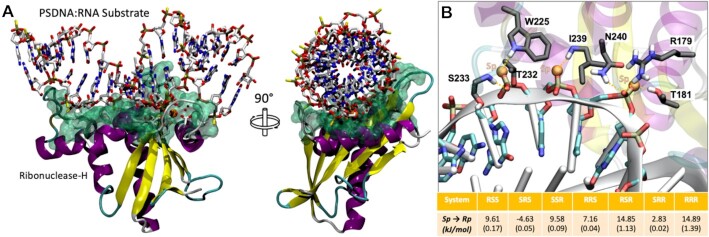
(**A**) Ternary complex of RNAse H in complex with SpDNA:RNA and catalytic Mg^2+^ ions. The highlighted green area refers to the interaction interface between the RNase H enzyme and the hybrid duplex. (**B**) Detail on the triad-substrate interactions and reported free energy data and relative standard errors in parenthesis. Motifs are reported in 3′-5′ direction.

The impact of phosphorothioate (presence and chirality) on RNase H activity is complex to predict, especially for the mammalian one, which has a convoluted recognition mechanism which involves contacts at both catalytic and hybrid binding domain (HBD). Thus, following Iwamoto *et al.* (23) we limited our attention to the catalytic domain, for which we have a high-resolution crystal structure that helped us to perform alchemical mutations between the eight possible diastereomers in the RNase H active site (see Materials and Methods and Supplementary Figure S13 for a general picture of the hybrid-protein complex). These, combined with the expected free energy changes in the unbound duplex (taken from our free-energy predictor; see above) led to approximated theoretical estimates of changes in binding free energies (Figure 6B). Interestingly, we observed a significant difference in binding energy, larger than what previously seen in folding free energies, which indicates that the effect of chirality in RNase H recognition is significant, not linear and complex. For example, substituting *SpSpSp* by *RpRpRp* (following Iwamoto's nomenclature we use here 3′-5′ orientation, see (Ref. 23) in RNAse H active site determines a remarkable destabilization of the binding to RNase H (+14.89 kJ/mol), which agrees with general claim that the *Sp* linkage confers a better RNase H susceptibility to the hybrid (28). However, the *scenario* is more complex and non-linear, as a single *Rp* surrounded by two *Sp* linkages turns to stabilize binding (Figure 6B) with respect to the *SpSpSp* diastereomer, and the *SpRpRp* combination leads to ∼4.5 kJ/mol better binding than the *RpRpSp*, even if the total ratio of *Rp/Sp* linkages is the same in both triads.

In order to validate these theoretical suggestions, we performed experiments using both *E. coli* and Human RNase H1 (including the HBD domain). Results shown in Supplementary Figure S14 (with details on the experiments) illustrates a good agreement with our theoretical estimates, supporting our claim that the *SpRpSp* triad is better for catalysis than the *SpSpRp* (always 3′-5′ direction); see details of the experiment in the label of Supplementary Figure S14.

## DISCUSSION

The impact of phosphorothioates (PS) in gene silencing approaches based on RNAse H degradation of DNA·RNA hybrids has been explored since the 90’s. Clearly, presence of PS linkages confers enzymatic stability to the DNA strand, leading to stable PS-DNA·RNA hybrids which are well processed by RNAse H. However, most of the experiments have been performed with racemic mixtures and studies focused on defining the different role of the two diastereomers (pro-*Sp* or pro-*Rp* oxygen substitution) have shown contrasting results, both in terms of thermal stability and susceptibility to RNAse H activity (15–30). Here, we present a massive computational effort (Force-Field Based Molecular Dynamic Simulations (MD), enhanced sampling techniques, QM/MM and statistical mechanics) complemented by a wealth of experimental measurements (thermodynamic analysis, circular dichroism and NMR spectroscopy), that allowed us to deliver physical-based predictive models on the effect of phosphorothioate stereochemistry on the structure, stability, flexibility and enzymatic susceptibility of PS-DNA and PS-DNA·RNA hybrids.

Spectroscopic studies and MD simulations confirm that structural changes induced by the presence of PS linkages in the DNA·RNA hybrid are moderate and the impact of PS chirality is mild, albeit significant. The *Sp* DNA·RNA hybrid assumes a more A-like conformation than the *Rp* DNA·RNA one that is instead closer to an A/B intermediate. The effect of PS chirality is local, as demonstrated by NMR and MD simulations of chimeras (*RpSp* and *SpRp*) (Supplementary Figures S3–S8), while transition from one chirality to the other introduces small structural changes located exclusively at the junction interface. DNA·RNA hybrids have a unique pattern of deformation compared to homopolymers, and a global flexibility similar to that of DNA·DNA for the first essential deformation modes and closer to that of RNA·RNA for the following ones. PS linkages makes hybrid stiffer, with pure *Rp* DNA·DNA hybrid showing a pattern of flexibility closer to that of the native DNA·RNA one, while essential deformations of the pure *Sp* hybrid are closer to those of the RNA·RNA duplex (Supplementary Figure S9 and Supplementary Table S1). The differences are however too modest to conclude that they might have a large impact in nuclease susceptibility.

*State-of-the-art* alchemical free energy calculations and accurate melting experiments suggest an order of stability: PO > *Rp* > *Sp* in agreement with Stec and co-workers (25,28,29), Wan *et al.* (30), and Iwamoto *et al.* (23), but in disagreement with claims from Ostergaard *et al.* (27), which might be related to a different experimental setup. The presence of *Sp* and *Rp* linkages alter hybrid stability in an additive manner, without significant (anti)cooperative effects. This, allowed us to develop a linear predictor based on the change in stability between native PO, *Rp* and *Sp* single linkage determined by theoretical free energy calculations (Figures 3 and 4), which shows an astonishing ability to reproduce experimental data (absolute error 3.8 kJ/mol, probably within accuracy of experimental estimates) on the relative stability of a variety of PO and PS hybrids, including chimeric ones obtained by mixing *Rp* and *Sp* diastereomers. The predictor can be used to determine the effect of phosphorothioates on the stability of any hybrid regardless their lengths and the chirality of the PS linkages.

The stability of PS DNA·RNA hybrids at physiological conditions is a key requirement for the biological applications of a PS-DNA single strand, but an effective ASO should be also resistant to exonucleases. MD and QM/MM calculations on the Klenow Fragment (47,84,85) strongly suggest that the presence of PS linkages distort the arrangement of catalytic residues at the nuclease active site, resulting in a predicted drop of the exonuclease activity. However, while such distortions are moderate for the *Rp* linkage, suggesting a reduction of exonuclease susceptibility, they resulted to be dramatic for the *Sp* one, which should be protected from exonuclease degradation. Experimental results corroborate the theoretical findings and show that conclusions could be also extrapolated to endonucleases such as DNAse I present in plasma (see Supplementary Figure S11)

A second requirement for PS-containing ASOs is that the resulting DNA·RNA hybrid should be degraded by RNAse H. Previous experiments suggested that indeed, DNA·RNA hybrids containing PS in the DNA strand are prone to get degraded by RNAse H with such a susceptibility depending to the chirality of the PS linkage (see Introduction). This is unexpected, since RNAse H hydrolyzes the RNA strand, the impact of chirality on the conformation of the RNA strand and in the dynamics of the hybrid described above is rather small as to justify chirality-dependent changes. This, suggests that the main impact of PS chirality on RNAse H susceptibility should be related to the change in affinity of the hybrid towards RNase H, and this must be reflected in changes in the interactions at the DNA-RNase H interface (Figure 6, Supplementary Figure S13). *Alchemical* free energy calculations performed on the hybrid-RNAse H complex (see Materials and Methods) and isolated hybrids strongly suggest a large and complex non-linear effect of PS chirality in hybrid recognition. In general *Sp* linkages are the best tolerated ones as experimentally described (28). However, rules are more complex, as a single *Rp* surrounded by two *Sp* linkages turns to stabilize binding (Figure 6B) with respect to the *SpSpSp* diastereomer, and the *SpRpRp* combination leads to 12 kJ/mol better binding than the *RpRpSp*, even the total ratio of *Rp/Sp* linkages is the same in both triads (for coherence with Iwamoto nomenclature 3′→5′ direction are used here) (23). Thus, our simulations support claims on the complexity and non-linearity raised by other authors (23,30,33), but not the claim on the optimum susceptibility conferred by the *SpSpRp* triad, as the *SpRpSp* triad seems to better recognise RNAse H enzyme. We can speculate the situation becoming even more complex in reality as the full-length RNAse H establishes more interactions with the DNA ASO backbone, which can be affected by the stereochemistry of the PS linkage.

The development of backbone modifications for ASO or siRNA drugs have been traditionally guided by serendipity and massive experimental efforts. We show here that combining structural studies and state-of-the-art simulation tools we can at least provide results that can guide the design of a new generation of chirality-controlled PS-based ASOs. Real *in vivo* activity is depending on many factors, some of which escape to theoretical simulations. However, any *in vivo* active oligonucleotide should: (i) lead to stable hybrids when paired with targeted RNA, (ii) resist to the action of serum nucleases, (iii) lead to hybrids sensitive to RNase H degradation. We show here that current simulation technology can be useful to investigate these points with reasonable accuracy, and find differences associated to minuscule changes in the backbone as is the R/S chirality of phosphate substitutions.

## DATA AVAILABILITY

The data that support the findings of this study are openly available in internal MMB servers at https://mmb.irbbarcelona.org/www/ reference name [PS-DNA]. https://mmb.irbbarcelona.org/BIGNASim/search.php?idQuickSearch=naked_, https://mmb.irbbarcelona.org/NAFlex/Vito/phosphorothioate.frcmod, https://mmb.irbbarcelona.org/NAFlex/Vito/phosphorothioate.lib.

## Supplementary Material

gkad309_Supplemental_File
