# Supplementary material for: Controlled sulfur-based engineering confers mouldability to phosphorothioate antisense oligonucleotides
Source: Nucleic Acids Res. 2023 Apr 26;51(10):4713–25. doi: 10.1093/nar/gkad309 (PMC10250214; doi:10.1093/nar/gkad309)
Supplement: gkad309_Supplemental_File [file gkad309_supplemental_file.pdf]

Controlled Sulfur-Based Engineering Confers Mouldability to Phosphorothioate Antisense Oligonucleotides

Vito Genna[1,2], Javier Iglesias[2], Laura Reyes-Fraile[1], Nuria Villegas[1], Kevin Guckian[3], Punit Seth[4], Brad Wan[4], Cristina Cabrero[5], Montserrat Terrazas[1,6], Isabelle Brun-Heath[1], Carlos González[5], Simone Sciabola[3], Anabella Villalobos[3] and Modesto Orozco[1,7]*

[1] Mechanisms of Diseases, Institute for Research in Biomedicine (IRB Barcelona), Baldiri Reixac 10-12, Barcelona, 08028, Spain

[2] NBD | Nostrum Biodiscovery, Baldiri Reixac 10, Barcelona, 08028, Spain

[3] Biogen, 225 Binney Street, Cambridge, MA, 02142, United States

[4] Ionis Pharmaceuticals Inc., 2855 Gazelle Court, Carlsbad, CA 92010, United States

[5] Instituto de Química Física Rocasolano, C/ Serrano 119, Madrid, 28006, Spain

[6] Department of Inorganic and Organic Chemistry, Section of Organic Chemistry, IBUB, University of Barcelona, Martí i Franquès 1-11, 08028 Barcelona, Spain

[7] Department of Biochemistry and Biomedicine, University of Barcelona, Barcelona, 08028, Spain

Supplementary Information

* To whom correspondence should be addressed.

Prof. Modesto Orozco
Tel: +34 93 40 37156
Email: modesto.orozco@irbbarcelona.org

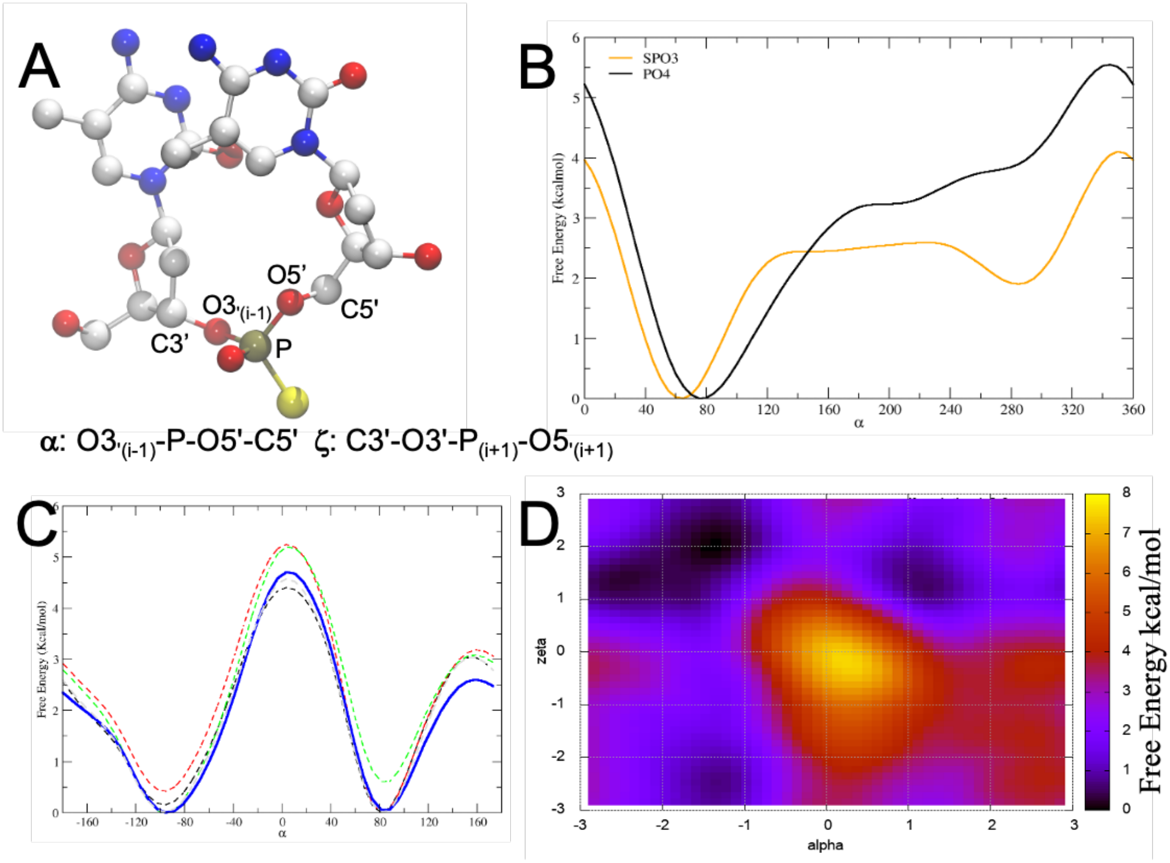

Figure S1. Parametrization of the α dihedral angle. **(A)** The modified DNA with thiophosphate substitution is reported. Dinucleotide has been considered to perform QM/MM parametrization of alpha dihedral angle. White refers to *C* atoms, red to *O*, Blue to *N*, Yellow to *S* while maroon to *P* atom. Atoms forming α and ζ dihedral angles are indicated. Picture has been realized with VMD.(1) **(B)** Potential Energy Surface (PES) of a-scanning in both *Sp*-modified DNA and wild-type one. Gaussian(2) software has been used to rotate the dihedral from 0 to 360 degrees (with a step size of 5 degrees) prior to minimize the rest of the structure. Hartree-Fock Method has been used to determine the wave function whit 6-31G* basis set. **(C)** Semi-empirical refinement using self-consistent-charge density-functional tight-binding method (SCC-DFTB).(3) Amber(4) and Terachem(5) have been used to generate trajectories. Dashed lines indicate the progressive convergence till the blue line (converged calculation). **(D)** Free energy Surface calculated to further check the reliability of DFT-based parametrization of a when coupled to the ParmBSC1 ζ-dihedral description.

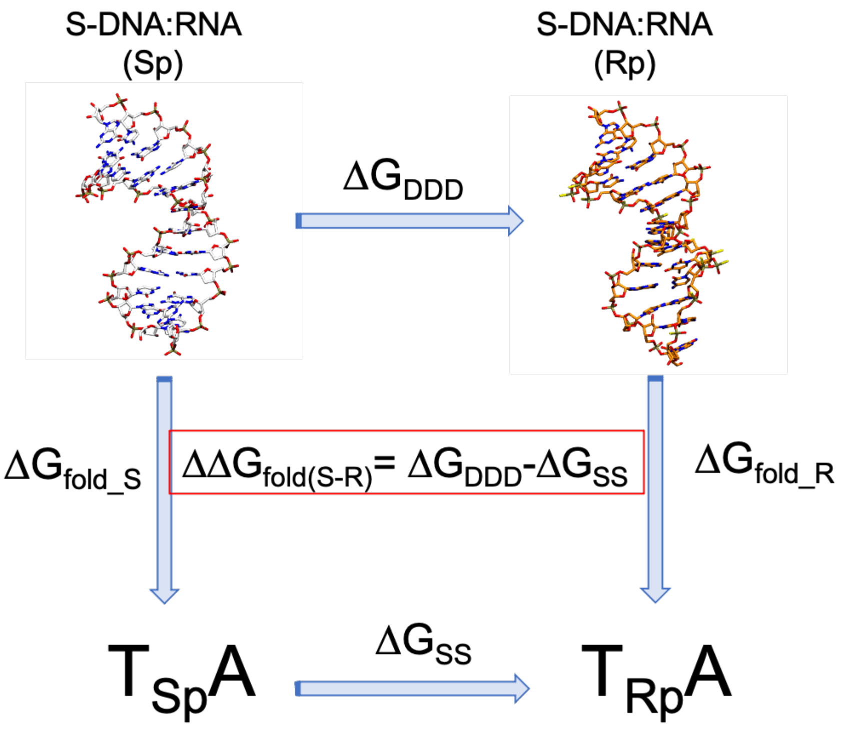

Figure S2. Explicative thermodynamic cycle adopted for all the alchemical transformations performed in this work. Please refer to the main text for detailed results.

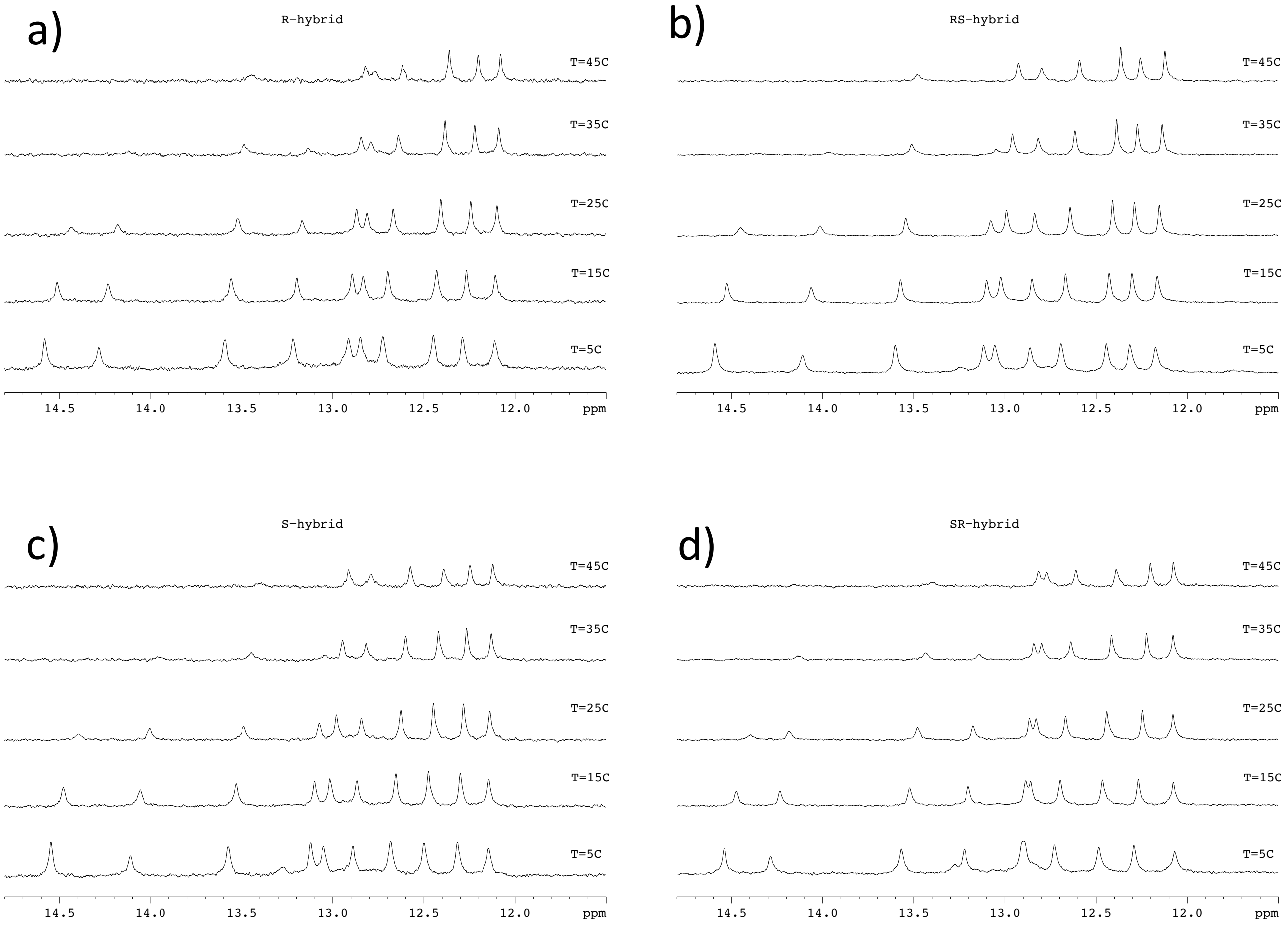

Figure S3. Imino proton regions of the ^1H NMR spectra of a) all *Rp*, b) *RpSp*, c) all *Sp* and d) *SpRp* duplexes in H_2O at different temperatures (100 mM NaCl 25 mM NaPi).

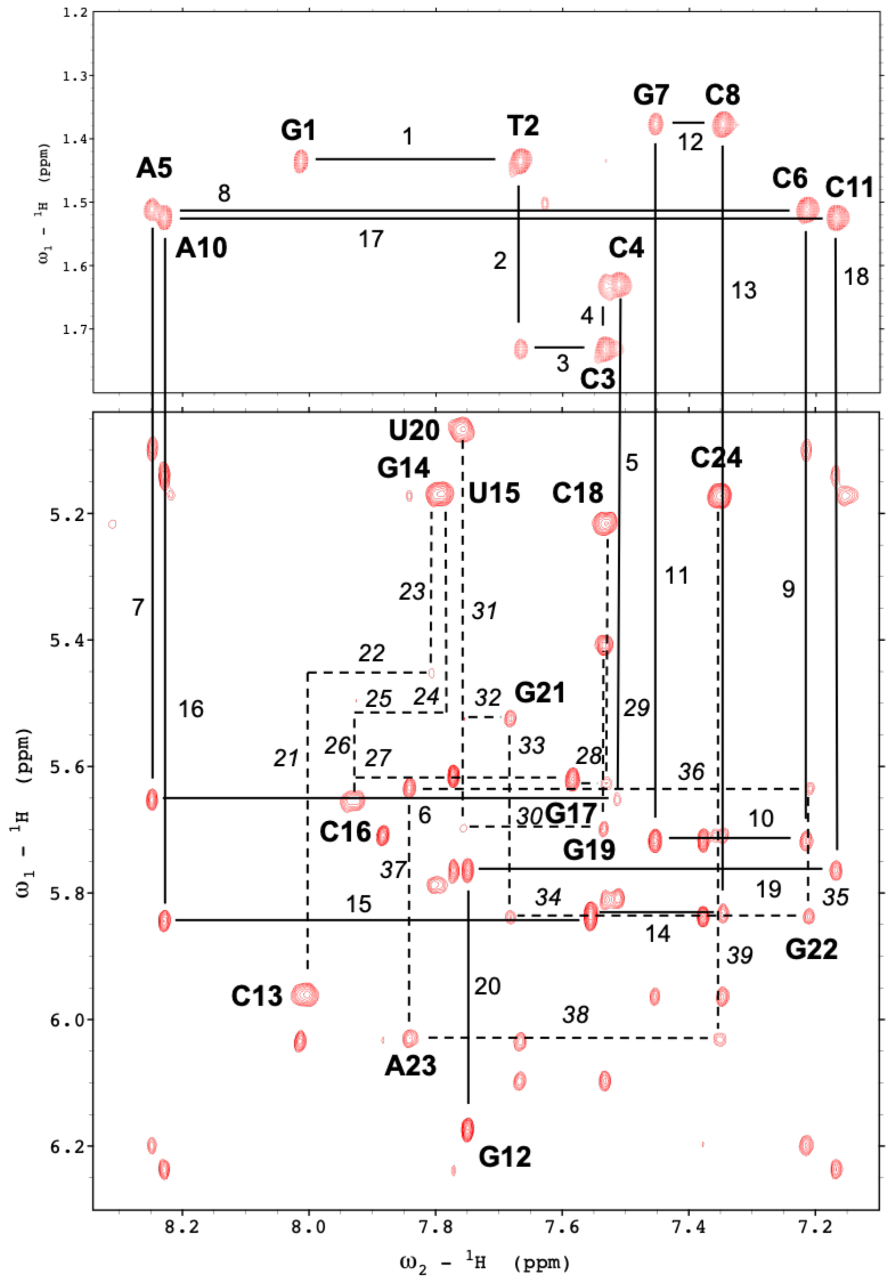

Figure S4. Region of the NOESY spectrum (250 ms mixing time) of all *Rp* [(GTC*C*AC*GC*GAC*G)-r(CAGGUGCGCUGC)] hybrid duplex in H_2O/ D_2O (500 uM oligonucleotide concentration, 100 mM NaCl 25 mM NaPi, T=5ºC, pH=7). Sequential assignment pathways for non-exchangeable protons are shown (RNA is shown with dashed lines).

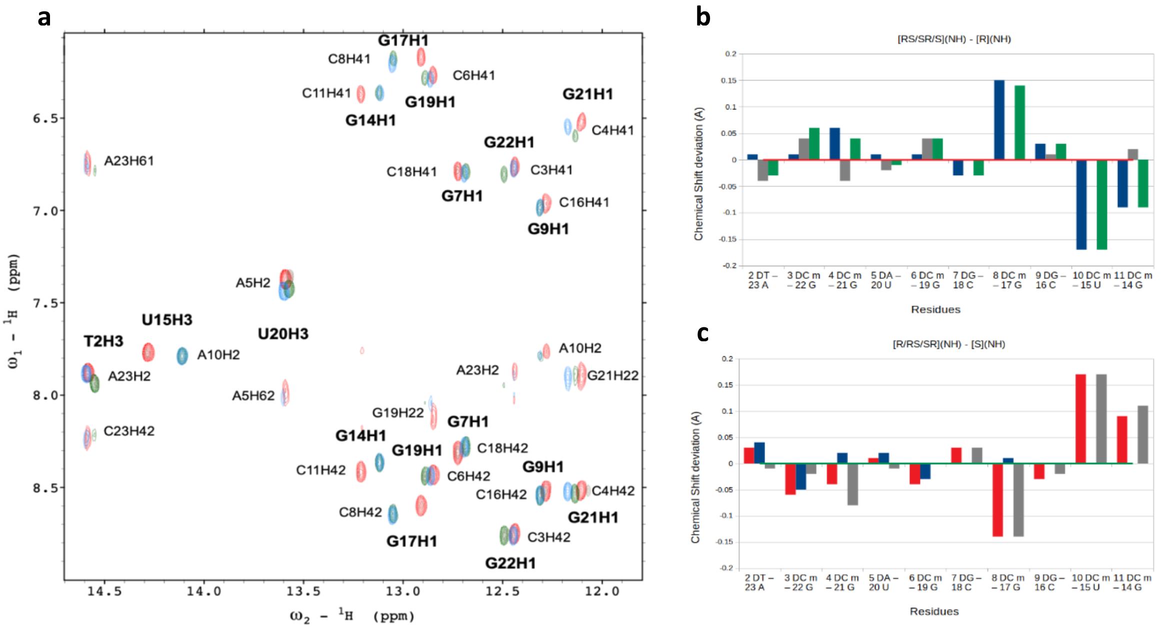

Figure S5. a) Superposition of the imino region of NOESY spectra in H_2O/ D_2O (t_m = 150ms) of *Rp* (red), *RpSp* (blue), all *Sp* (green) and *SpRp* (gray). Chemical shift difference of all the hybrids with respect to *Rp* (b) and *Sp* (c). Same color code as the spectra. Buffer conditions were 100 mM NaCl 25 mM NaPi, T= 5 ºC, pH 7.

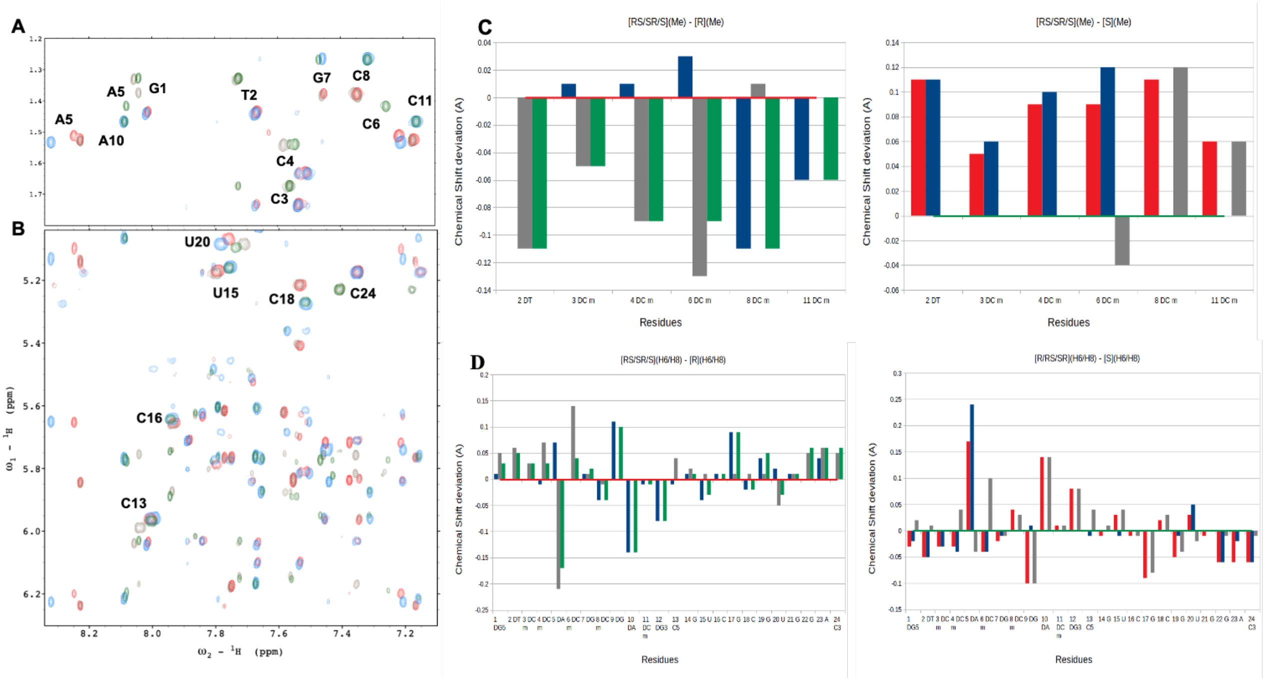

Figure S6. Spectral comparison of aromatic and methyl protons. Methyl (A) and aromatic (B) regions of the NOESY spectra (250ms mixing time) of all *Rp* (red), *RpSp* (blue), all *Sp* (green) and *SpRp* (gray). c) Chemical shift differences of methyl groups with respect to all *Rp* (left) and all *Sp* (right). D) Chemical shift differences of aromatic (H6/H8) protons with respect to all *Rp* (left) and all *Sp* (right). Chemical shift difference of all the hybrids with respect to *Rp* (b) and *Sp* (c). Same color code as in Figure S5. Buffer conditions were 100 mM NaCl 25 mM NaPi, T=5ºC, pH 7.

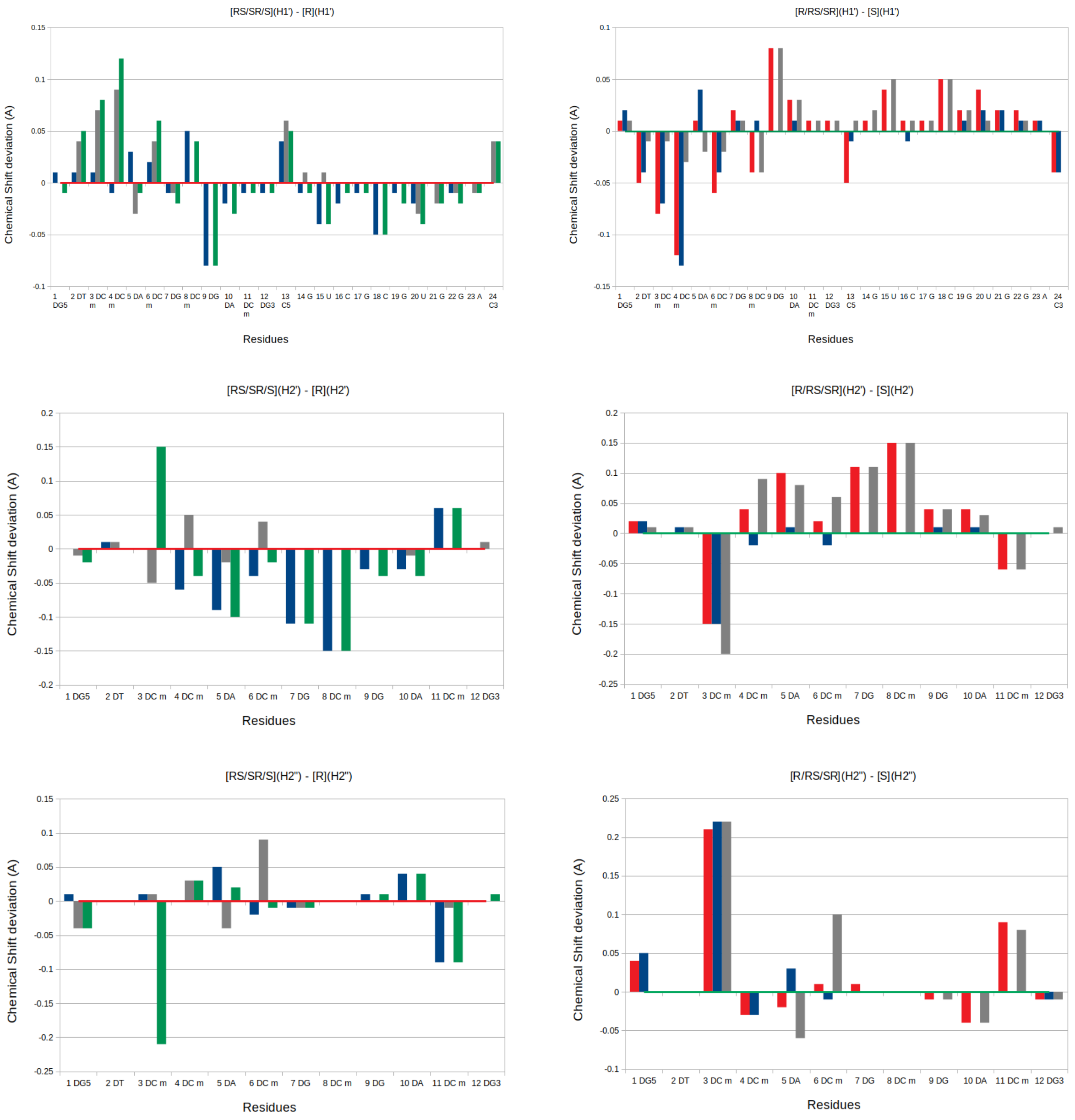

Figure S7. Spectral comparison of sugar protons. Chemical shift differences of sugar protons, H1'(first row), H2'(second row) and H2"(third row), of the four hybrids with respect to *Rp* (left) and *Sp* (right). Same color code and buffer conditions as in Figure S5.

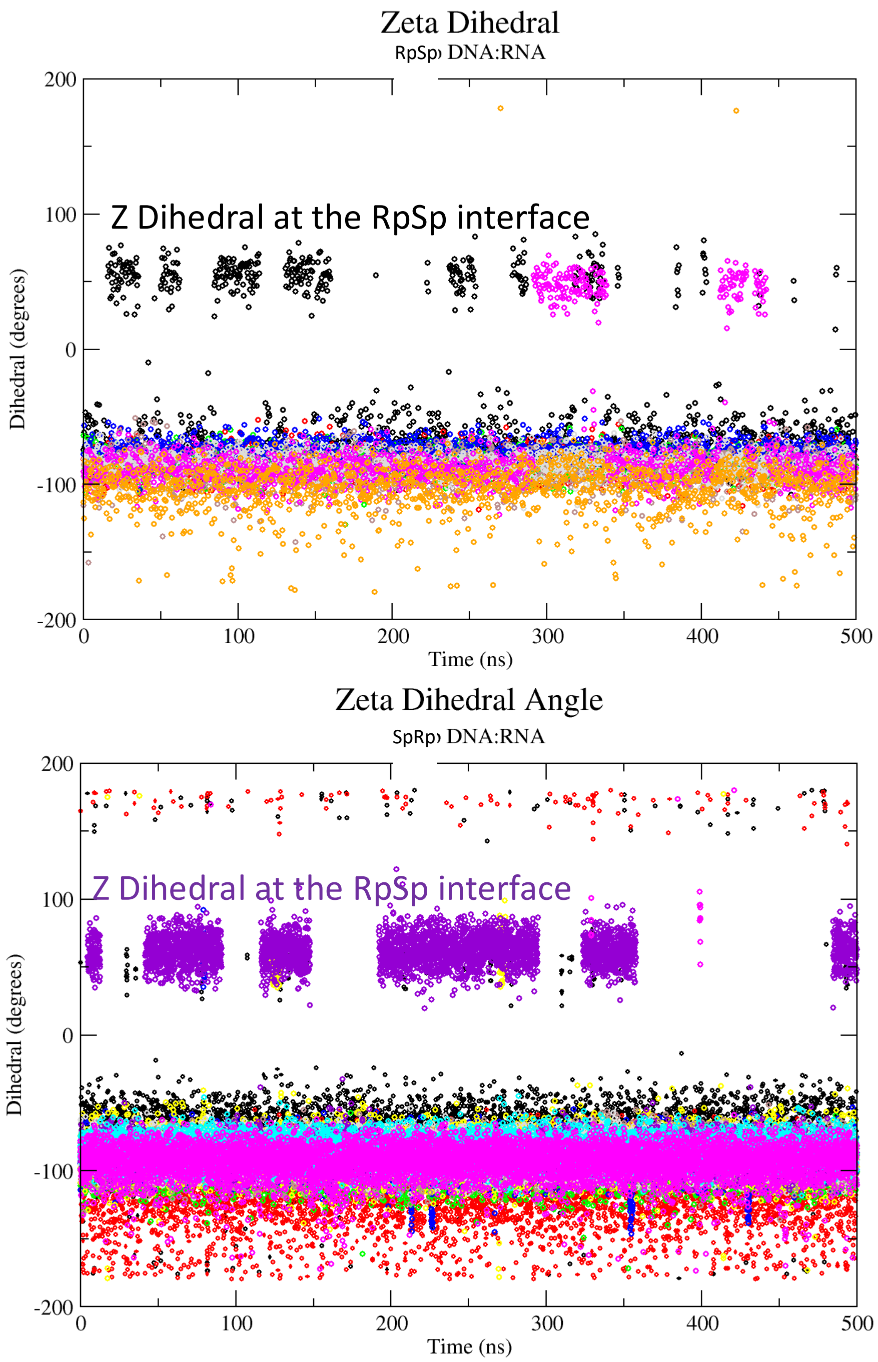

Figure S8. Plot of the ζ-dihedral angles (i.e., C3'-O3'-P-O5') along the time for the *SpRp* and *RpSp* systems.

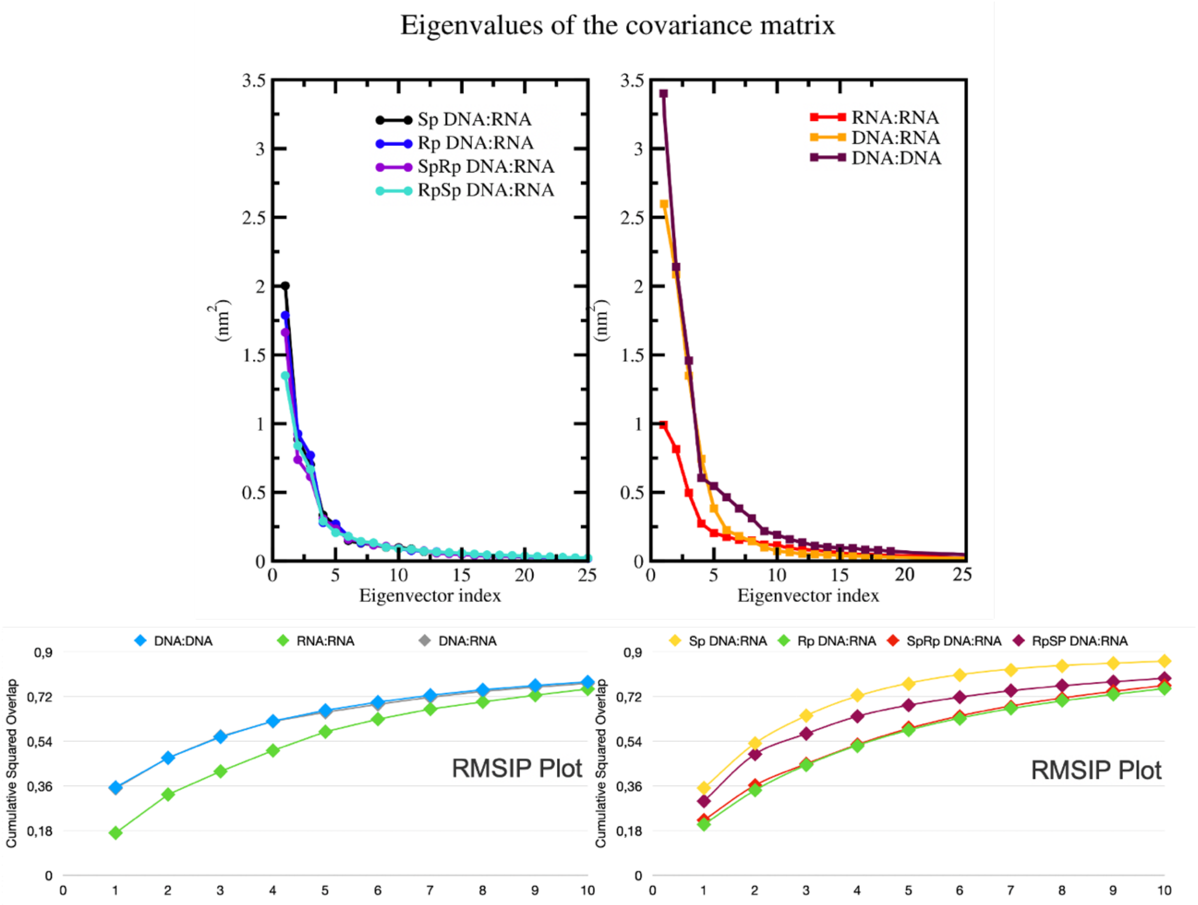

Figure S9. Eigenvalues of the first 25 eigenvectors of calculated for the systems under investigation. Upper right panel reports the reference systems (DNA:DNA, RNA:RNA and DNA:RNA). Covariance matrix was calculated between each single trajectory and an average structure of it. Selection includes the following atoms: P, OP1 (or S), OP2 (or S), O3', C3', C2', C1', O4', C4', C5' and O5' for a total of 220 atoms. Terminal base pairs were removed to avoid noise. Backbone selection has been maintained constant for each single calculation. In the bottom graph, root mean square inner product (RMSIP) is reported for the first 10 eigenvectors.

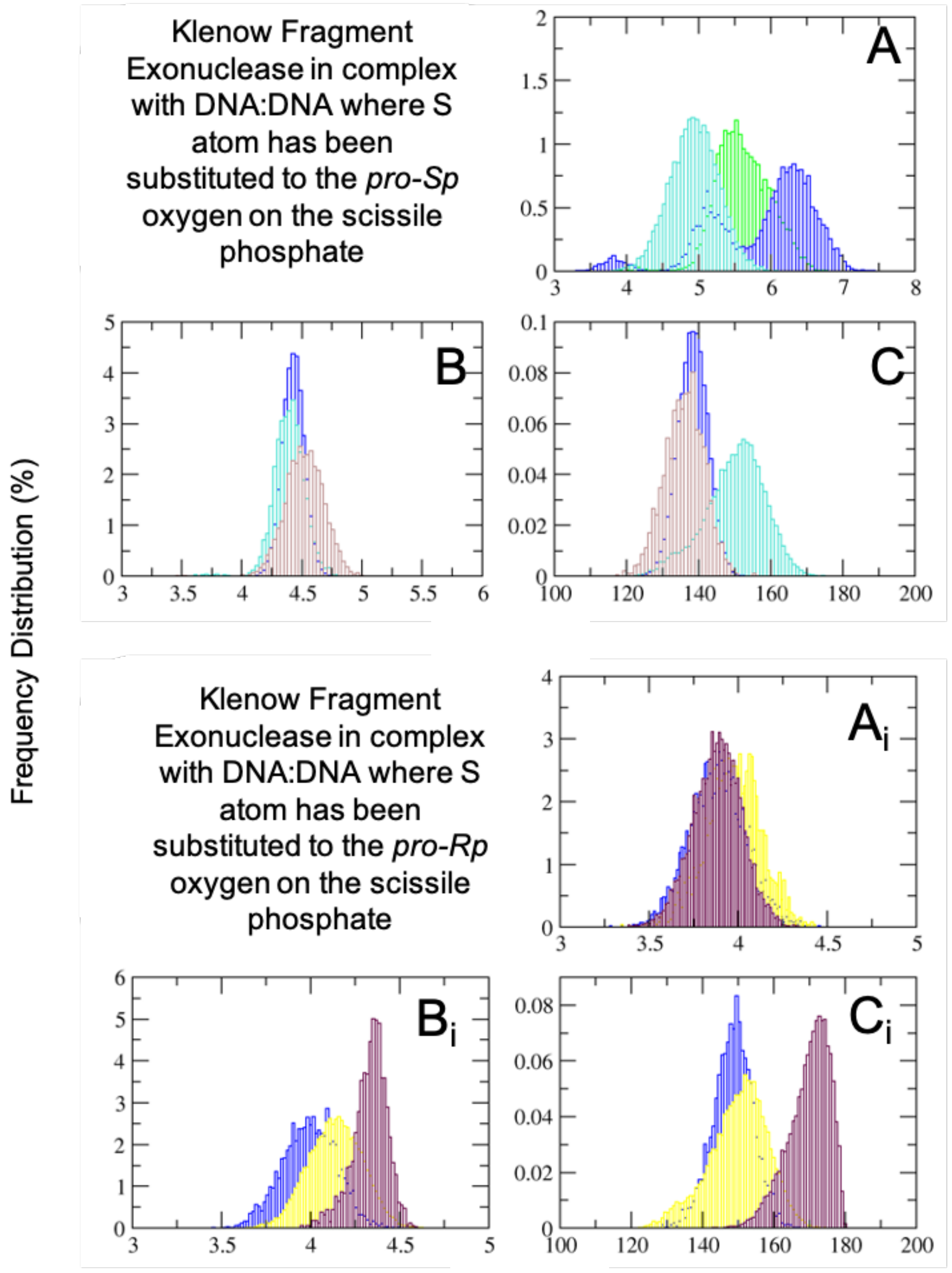

Figure S10. Structural comparison between *pro-Sp* and *pro-Rp* S-substituted phosphate in Klenow Fragment Exonuclease (PDBid 1KFS). Each system has been simulated for ~100 ns (production runs only has been taken into account for the present measurements). Different colours identify different replicas. Letter **A** indicates the reaction coordinate length (i.e., $_{d}$Nuc see Figure 5 in the main text) in Å (shorter the distance easier the reaction), letter **B** shows the Mg^{2+}-Mg^{2+} internuclear length (in Å), shorter values indicate a better prone-to-react complex. Letter **C** indicate the value of the S_N2-angle (i.e., along the reactive atoms O-P-O), X-axis reports angle degree (closer to 180 degrees better the alignment).

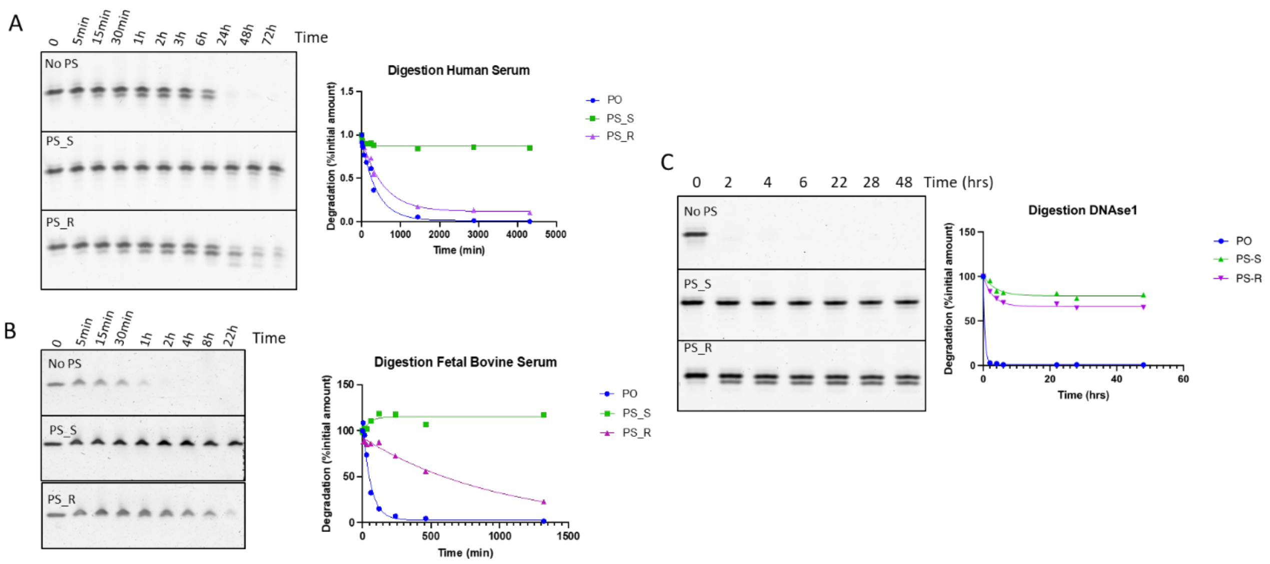

Figure S11. Serum and endonuclease digestion. 200pmoles of DNA oligonucleotide were incubated at 37ºC with 50% Human serum (A), with 55% Fetal bovine serum (B) or with 0.5 unit of Bovine DNAse 1 (C). After heating at 95ºC, samples were analyzed by 20% Urea-PAGE. For each panel, the control with phosphodiester bonds (No PS) is on top, the oligonucleotide with Sp phosphorothioate bonds (PS_S) in the middle and the one with Rp phosphorothioate bonds (PS_R) at the bottom.

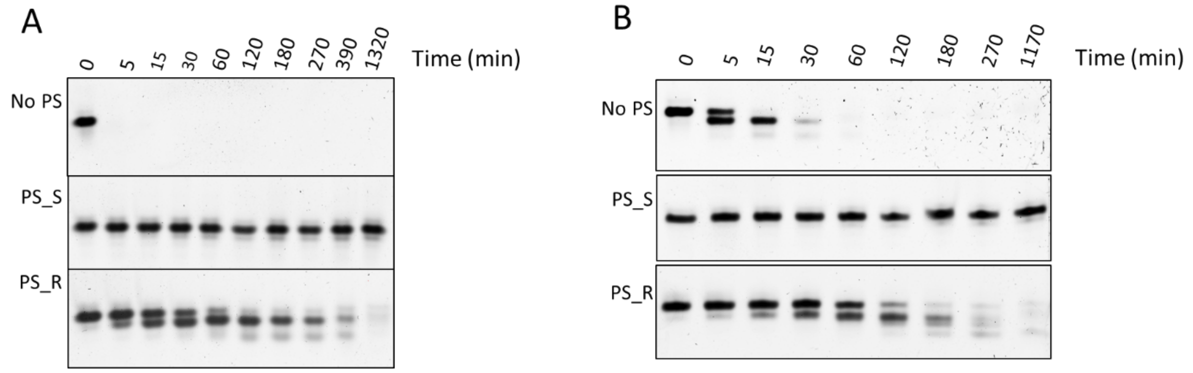

Figure S12. Exonuclease digestion. 200pmoles of DNA oligonucleotide were incubated at 37ºC with 50 mUnits of Snake Venom Phosphodiesterase 1 (A) or 10 Units of Klenow fragment (B). After heating at 95ºC, samples were analyzed by 20% Urea-PAGE. For each panel, the control with phosphodiester bonds (No PS) is on top, the oligonucleotide with Sp phosphorothioate bonds (PS_S) in the middle and the one with Rp phosphorothioate bonds (PS_R) at the bottom.

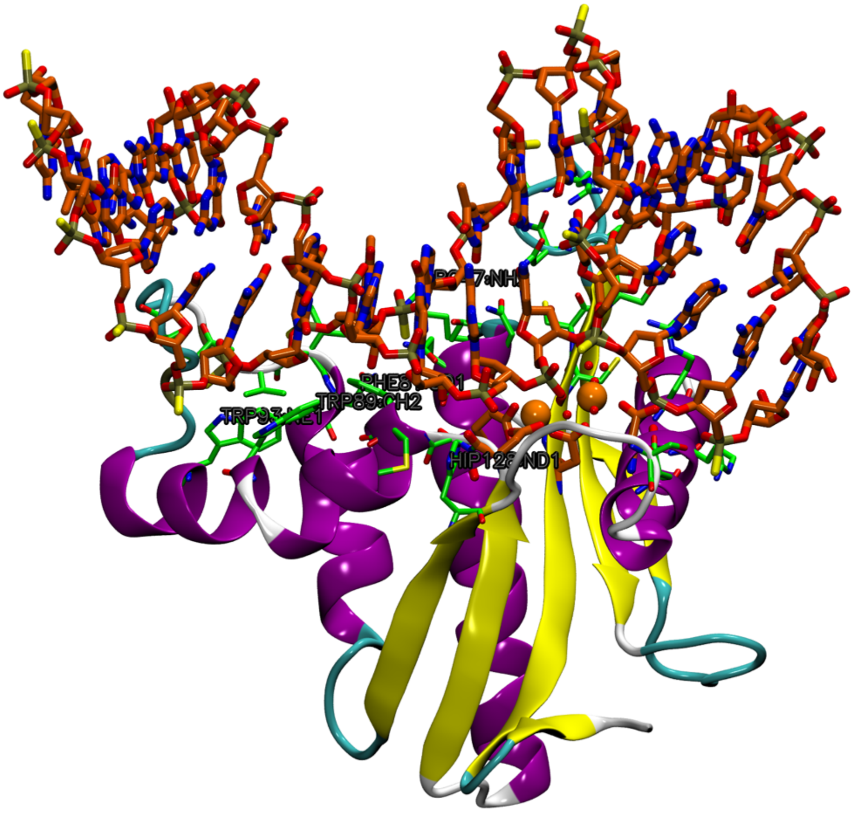

Figure S13. RNAse-H in complex with *Sp* DNA:RNA Hybrid. Protein is shown in cartoon while DNA:RNA substrate in licorice (orange carbons). Protein residues reported in green refer to those falling in a 3 Å coordination sphere from the DNA:RNA substrate. The poor protein-substrate interface is mainly formed by aromatic amino acids which, by establishing hydrophobic interactions with the sugars of the substrate, keep well-posed the double helix on the RNase H hosting cleft. Other alkylic side-chain play a role in stabilizing the substrate by forming H-bond interaction with polar species present in the major and minor grove of the substrate; mainly found closeby the active site (identified by the presence of the two orange spheres referring to Mg^{2+} ions).

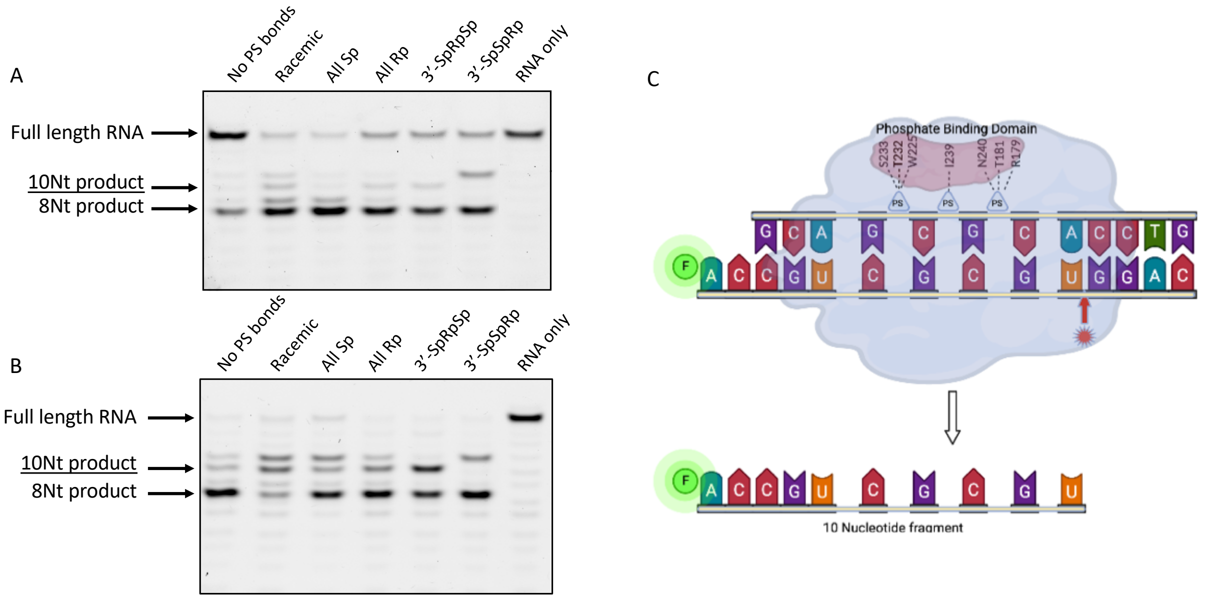

Figure S14. RNase H1 cleavage of 5' fluorescently labeled RNA in DNA PS/RNA duplexes ((dGCAG**<u>CGC</u>**ACCTG)·r(CAGGUGCGUUGCCA-FAM), with changes made in the central motif CGC). 5pmoles of DNA-RNA duplex were digested with of 13.5ng of human RNAse H1 during 4 hrs at 37ºC (A) or 50pmoles of DNA-RNA duplex were digested with 1u of *E. coli* RNAse H during 15 min at 37ºC (B). After heating at 95ºC, samples were analyzed by 20% Urea-PAGE. C) Schematic representation of RNase H1 cleavage when the 3 phosphorothioate bonds are in the Phosphate Binding Domain.

	DNA:DNA	RNA:RNA	DNA:RNA	Sp DNA:RNA	Rp DNA:RNA	SpRp DNA:RNA	RpSp DNA:RNA
DNA:DNA	0.93	0.52	0.61	0.63	0.59	0.65	0.67
RNA:RNA		0.95	0.60	0.72	0.60	0.56	0.57
DNA:RNA			0.92	0.62	0.78	0.71	0.72
Sp DNA:RNA				0.90	0.76	0.73	0.74
Rp DNA:RNA					0.90	0.73	0.74
SpRp DNA:RNA						0.94	0.81
RpSp DNA:RNA							0.93

Supplementary Tab S1. Dot products between essential modes. Grey boxes contain values for self-similarity index (convergence check). Please Refer to the main text for further details.

(A)

Hybrid duplexes studied in this work	Tm at 0.1mM	ΔHº (kJ/mol)	ΔS º(kJ/mol/K)	ΔGº$_{37ºC}$(kJ/mol)
**d(GTCCACGCGACG)· r(CGTCGCGTGGAC)**	76.38	-424.93	-1.13	-75.19 +/- 0.55
**d(*GTCCACGCGACG*)· r(CGTCGCGTGGAC)**	64.60	-485.27	-1.06	-59.09 +/- 0.24
**d(GTCCACGCGACG)· r(CGTCGCGTGGAC)**	71.74	-406.70	-1.09	-68.25 +/- 0.45
**d(GTCCA*CGCGACG*)· r(CGTCGCGTGGAC)**	67.87	-385.53	-1.03	-62.31 +/- 1.43
**d(*GTCCA*CGCGACG)· r(CGTCGCGTGGAC)**	68.34	-397.94	-1.07	-63.68 +/- 1.19

(B)

Hybrid duplexes studied in this work	Tm at 0.1mM	ΔHº (kJ/mol)	ΔS º(kJ/mol/K)	ΔGº$_{37ºC}$(kJ/mol)
**d(GTCCACGCGACG)· r(CGTCGCGTGGAC)**	76.38	-101.56	-0.27	-17.96 +/- 0.1
**d(*GTCCACGCGACG*)· r(CGTCGCGTGGAC)**	64.60	-115.98	-0.25	-14.12 +/- 0.0
**d(GTCCACGCGACG)· r(CGTCGCGTGGAC)**	71.74	-97.20	-0.26	-16.31 +/- 0.1
**d(GTCCA*CGCGACG*)· r(CGTCGCGTGGAC)**	67.87	-92.14	-0.25	-14.87+/- 0.3
**d(*GTCCA*CGCGACG)· r(CGTCGCGTGGAC)**	68.34	-95.11	-0.26	-15.26 +/- 0.3

Supplementary Tab S2. (A) Melting Temperature (in Celsius), Entropy, Enthalpy and folding free energies for different hybrids determined experimentally. Values obtained by averaging the results from the three methods implemented in MeltR (6) to fit absorbance melting curves (standard deviations are shown for the absolute free energy of folding. Roman means standard phosphate linkages, underlined characters stand for the R PS linker (Rp) and italics refers to S PS linker (Sp). (B) In order to facilitate reading of the Table the same results in kcal/mol units.

References

1. Humphrey, W., Dalke, A. and Schulten, K. (1996) VMD: visual molecular dynamics. *J Mol Graph*, **14**, 33-38, 27-38.
2. Frisch, M.J.T., G. W.; Schlegel, H. B.; Scuseria, G. E.; Robb, M. A.; Cheeseman, J. R.; Scalmani, G.; Barone, V.; Mennucci, B.; Petersson, G. A.; Nakatsuji, H.; Caricato, M.; Li, X.; Hratchian, H. P.; Izmaylov, A. F.; Bloino, J.; Zheng, G.; Sonnenberg, J. L.; Hada, M.; Ehara, M.; Toyota, K.; Fukuda, R.; Hasegawa, J.; Ishida, M.; Nakajima, T.; Honda, Y.; Kitao, O.; Nakai, H.; Vreven, T.; Montgomery, J. A., Jr.; Peralta, J. E.; Ogliaro, F.; Bearpark, M.; Heyd, J. J.; Brothers, E.; Kudin, K. N.; Staroverov, V. N.; Kobayashi, R.; Normand, J.; Raghavachari, K.; Rendell, A.; Burant, J. C.; Iyengar, S. S.; Tomasi, J.; Cossi, M.; Rega, N.; Millam, J. M.; Klene, M.; Knox, J. E.; Cross, J. B.; Bakken, V.; Adamo, C.; Jaramillo, J.; Gomperts, R.; Stratmann, R. E.; Yazyev, O.; Austin, A. J.; Cammi, R.; Pomelli, C.; Ochterski, J. W.; Martin, R. L.; Morokuma, K.; Zakrzewski, V. G.; Voth, G. A.; Salvador, P.; Dannenberg, J. J.; Dapprich, S.; Daniels, A. D.; Farkas, Ö.; Foresman, J. B.; Ortiz, J. V.; Cioslowski, J.; Fox, D. J. (2009) Gaussian 09, Revision D.01. *Wallingford CT*.
3. Gaus, M., Cui, Q. and Elstner, M. (2012) DFTB3: Extension of the self-consistent-charge density-functional tight-binding method (SCC-DFTB). *J Chem Theory Comput*, **7**, 931-948.
4. Case, D.A., Cheatham, T.E., 3rd, Darden, T., Gohlke, H., Luo, R., Merz, K.M., Jr., Onufriev, A., Simmerling, C., Wang, B. and Woods, R.J. (2005) The Amber biomolecular simulation programs. *J Comput Chem*, **26**, 1668-1688.
5. Liu, F., Luehr, N., Kulik, H.J. and Martinez, T.J. (2015) Quantum Chemistry for Solvated Molecules on Graphical Processing Units Using Polarizable Continuum Models. *J Chem Theory Comput*, **11**, 3131-3144.
6. Sieg, J. P., McKinley, L. N.; Huot, M. J., Yennawar, N. H. and Bevilacqua, P. C. (2022) The Metabolome Weakens RNA Thermodynamic Stability and Strengthens RNA Chemical Stability. *Biochemistry*, **61**, 22, 2579-2591.
